# piENOX2 regulates ALKBH5-mediated *Itga4* m^6^A modification to accelerate the progression of rheumatoid arthritis

**DOI:** 10.1038/s12276-025-01503-3

**Published:** 2025-07-23

**Authors:** Naibo Feng, Chungeng Liu, Yuan Zhu, Shuqiong Cai, Yongheng Xie, Zhenmin Wang, Hua Wang, Guozhi Xiao, Houqing Long, Songlin Peng

**Affiliations:** 1 Division of Spine, Department of Orthopedic Surgery, Shenzhen People’s Hospital, The Second Clinical Medical College, Jinan University; The First Affiliated Hospital, Southern University of Science and Technology, Shenzhen, China; 2 Department of Trauma Orthopedics, Weifang People’s Hospital, Shandong Second Medical University, Weifang, China; 3 Department of Cardiovascular Surgery, Guangdong Cardiovascular Institute, Guangdong Provincial People’s Hospital, Guangdong Academy of Medical Sciences, Southern Medical University, Guangzhou, China; 4 Department of Joint Surgery, Longhua Branch, Shenzhen People’s Hospital, The Second Clinical Medical College, Jinan University; The First Affiliated Hospital, Southern University of Science and Technology, Shenzhen, China; 5 School of Medicine, Southern University of Science and Technology, Guangdong Provincial Key Laboratory of Cell Microenvironment and Disease Research, Shenzhen Key Laboratory of Cell Microenvironment, Shenzhen, China

**Keywords:** Autoimmunity, Rheumatoid arthritis, Autoimmune diseases

## Abstract

Rheumatoid arthritis (RA) is a chronic autoimmune disorder characterized by synovitis and presenting as symmetrical arthritis that primarily affects the small joints of the limbs. PIWI-interacting RNAs, a class of small noncoding RNAs, have garnered significant attention due to their critical involvement in various pathological conditions, including reproductive diseases, cancers and other disorders. Here we observe elevated levels of macrophage-derived piENOX2 in the synovial tissues of both patients with RA and mice with collagen-induced arthritis (CIA). It was found that transfection with a piENOX2 mimic promoted M1 macrophage polarization, while a piENOX2 inhibitor facilitated M2 polarization. In vivo, a piENOX2 inhibitor significantly alleviated disease progression, reduced systemic inflammation and preserved the integrity of articular cartilage in CIA mice. Mechanistic analyses indicated that the piENOX2 effects were due to its targeting *Alkbh5* mRNA for degradation. In a *Alkbh5* conditional-knockout mouse model of CIA, the therapeutic effects of a piENOX2 inhibitor, including inflammation suppression and cartilage protection, were reduced compared with control mice. A comprehensive analyses using methylated RNA immunoprecipitation sequencing and methylated RNA immunoprecipitation and quantitative PCR revealed that piENOX2 regulated ALKBH5-mediated m^6^A modification of *Itga4* mRNA, thereby influencing macrophage polarization through the PI3K–AKT signaling pathway. These findings provide important insights into the complex roles of PIWI-interacting RNAs in RA progression and indicate potential avenues for therapeutic intervention.

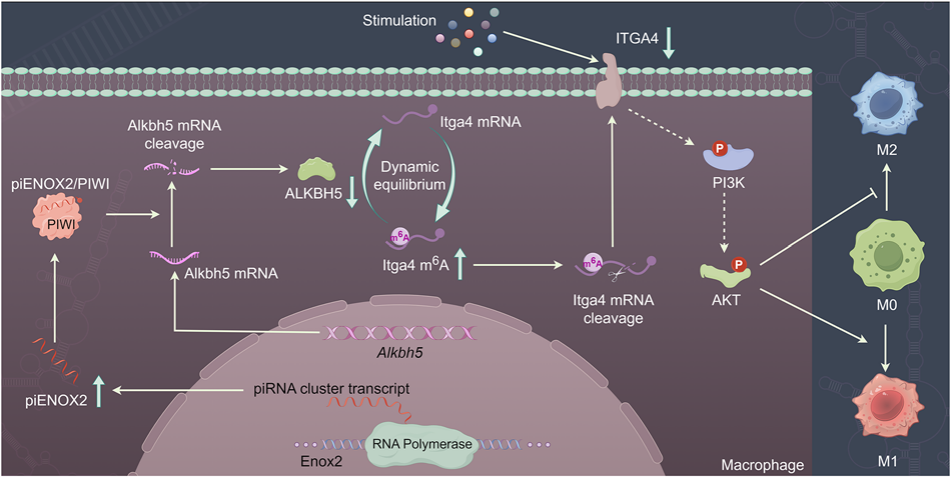

## piENOX2 modulates macrophage polarization in rheumatoid arthritis

Rheumatoid arthritis is a chronic disease where the immune system attacks the joints, causing pain and damage. Researchers studied a small RNA molecule called piENOX2, which is found in higher levels in joint tissues of patients with rheumatoid arthritis. They discovered that piENOX2 affects immune cells called macrophages, promoting inflammation. Researchers used a mouse model of rheumatoid arthritis to test a new treatment targeting piENOX2. They created a drug delivery system using liposomes to carry a piENOX2 inhibitor directly to macrophages. This treatment reduced joint damage and inflammation in the mice. The study found that piENOX2 influences macrophage behavior by affecting a gene called Alkbh5, which in turn impacts another gene, *Itga4*, involved in cell signaling pathways. By blocking piENOX2, the treatment helped shift macrophages from a proinflammatory state to an anti-inflammatory one.

This summary was initially drafted using artificial intelligence, then revised and fact-checked by the author.

## Introduction

Rheumatoid arthritis (RA) is a chronic autoimmune condition characterized by synovitis and joint damage, leading to deformities and functional impairment^1^. Activated synovial macrophages are known to promote inflammation and tissue damage. Current RA drug interventions face challenges, including complications and relapses, indicating the need for novel drug targets and a deeper understanding of RA pathogenesis^2^.

The emergence of mRNA vaccines during the coronavirus disease 2019 pandemic has spurred interest in research on small nucleic acid drugs. Small nucleic acids such as small interfering RNAs (siRNAs), and antisense oligonucleotides show promise for RA therapeutics. PIWI-interacting RNA (piRNA), discovered in the year 2006^3–6^, regulates transposable elements, epigenetic modifications and protein modulation through the piRNA/PIWI complex^7,8^. Dysregulated piRNA function is implicated in condition, asthenozoospermia and male infertility^9–11^. Furthermore, abnormal expression of piRNAs is seen in various tumor tissues, exerting either promotive or suppressive influences on cancer and associated closely with tumor classification and staging^12–16^. Significantly, perturbations in the piRNA/PIWI pathway feature prominently in neurodevelopmental disorders and neurodegenerative diseases^17–19^. Our previous preliminary investigations observed marked upregulation of piR-63049 in bone tissue and plasma samples from osteoporotic rats and postmenopausal patients with osteoporosis. Notably, piR-63049 overexpression impedes the osteogenic differentiation of bone marrow stromal cells, while piR-63049 knockdown promotes osteogenic differentiation via the Wnt2b/β-catenin signaling pathway. This novel piRNA thus has potential as a target for treating disorders associated with bone loss^20^. Although piRNAs are associated with various diseases, their role in the pathogenesis of RA remains largely unexplored.

In this study, small-RNA sequencing and flow cytometry revealed that macrophage-derived piENOX2 is overexpressed in RA synovial tissue and mice with collagen-induced arthritis (CIA). In vitro experiments demonstrated that piENOX2 plays a critical regulatory role in macrophage polarization. Moreover, a macrophage-targeted piENOX2 inhibitor liposomal prodrug was designed and synthesized, and its efficacy in cartilage protection, immunomodulation and inflammation control was verified in CIA mice. Using gene-deficient mice and methylated RNA immunoprecipitation (meRIP) sequencing (meRIP-seq), an in-depth mechanistic exploration revealed that piENOX2 regulated macrophage polarization by modulating ALKBH5-mediated *Itga4* m^6^A modification and inhibiting the PI3K–AKT signaling pathway, thereby contributing to RA progression. These findings clarify the mechanistic role of piENOX2 and identify new therapeutic targets for RA.

## Materials and methods

### DAS-28 assessment

The Disease Activity Score in 28 joints (DAS-28) is a validated composite tool for assessing RA disease activity, combining: (1) tender joint count (TJC28); (2) swollen joint count (SJC28), evaluated across 28 joints (shoulders, elbows, wrists, metacarpophalangeals, proximal interphalangeals and knees); (3) erythrocyte sedimentation rate (in millimeters per hour); and (4) patient global health (GH): self-reported health status on a 100-mm visual analog scale.

The DAS-28 score is calculated using the following formula: ${\rm{DAS}}{-}28=0.56\times \sqrt{{{{TJC}}}28}+0.28\times \sqrt{{{{SJC}}}28}+0.70\times {\mathrm{ln}}({\rm{ESR}})+0.014\times {\rm{GH}}.$

### CIA model

CIA was induced in 6–8-week-old male DBA1/J mice. Complete Freund’s adjuvant (Sigma-Aldrich, cat. no. F5881) was added to an equal volume of 2 mg ml^−1^ bovine type II collagen (Chondrex, cat. no. 20022) on ice and emulsified using a homogenizer, taking care to prevent heat-induced denaturation. The mice were anesthetized with 0.1 ml of the emulsion was injected intradermally at two points. A booster with an emulsion of bovine type II collagen in incomplete Freund’s adjuvant (Sigma-Aldrich, cat. no. F5506) was given on day 21. The control mice were injected with physiological saline only.

### piRNA sequencing

A total of 2 weeks after booster administration, the mice were euthanized by cervical dislocation, and synovial tissue from the knee joints was collected, flash-frozen in liquid nitrogen and sent for sequencing. The sequencing procedure included total RNA extraction, reverse transcription, PCR amplification and library construction. Sequencing was performed on an Illumina HiSeq 2500 platform. The raw reads were filtered, yielding clean reads, which were compared with the reference genome to create a read distribution map. The clean reads were annotated for noncoding RNA classification. The expression levels, clustering and differential expression of identified piRNAs were determined. The predicted piRNA target genes were analyzed by Gene Ontology (GO) and Kyoto Encyclopedia of Genes and Genomes (KEGG) enrichment analyses.

### FISH

Fluorescence in situ hybridization (FISH) fluorescent probes and kits were custom designed and supplied by Shanghai GenePharma. Synovial tissue samples were collected from patients with RA and control subjects, fixed and sectioned. Subsequent to sample preprocessing, as per the guidelines provided by the reagent manufacturer, the sections were subjected to incubation with the Cy3-labeled piENOX2 fluorescent probe overnight at 4 °C, in the dark. Following thorough washing, DAPI staining was applied and post-sealing, the fluorescence expression and distribution were observed and imaged under fluorescence microscopy.

### piRNA screening

All protocols involving human specimens were approved by the Ethics Committee of Shenzhen People’s Hospital. Synovial tissue specimens were obtained following surgical resection, and all patients provided informed consent. The inclusion criteria were RA diagnosed according to the 1987 revised classification criteria of the American College of Rheumatology, with surgical invention due to significant pain or deformity. Individuals with other joint disorders, severe cardiac dysfunction, chronic diseases or inability to tolerate general anesthesia and surgery were excluded. The controls were resected specimens from patients with osteoarthritis.

Mouse and human piRNA sequences were compared using piRBase. The top ten most significantly upregulated and downregulated piRNAs were selected, and the primers were designed and synthesized. Total RNA was extracted from synovial tissues and piRNA expression was evaluated by reverse transcription quantitative PCR (RT–qPCR), identifying piRNAs consistent between the sequencing and RT–qPCR. Mimics and inhibitors for these piRNAs were designed and synthesized by Guangzhou RiboBio. The selected piRNAs were further analyzed in transfected lipopolysaccharide (LPS; Sigma-Aldrich, cat. no. L5293)-treated cells to assess levels of IL-1β, IL-6 and TNF.

### Tissue collection and flow cytometry

The CIA mouse model was established following standard protocols. At specified time points, mice were killed and immersed in 75% ethanol for 10 min, and the synovial tissues were collected. The location of the right hind-knee patellar tendon was identified, and adjacent muscle tissue was carefully dissected enabling exposure of the quadriceps tendon. A small incision was made above the patella on the quadriceps tendon, and fine forceps were used to expose the joint capsule attached to the femur, enabling dissection of the joint capsule along the femur and tibia to collect the synovial tissue. Moderate traction was applied to the quadriceps tendon during dissection, pausing when only the synovial tissue attached to the tibia remained. The infrapatellar fat pad was separated, and the remaining joint capsule was incised to extract the synovial tissue.

The synovial tissue was cut into small pieces and placed in RPMI 1640 (Gibco, cat. no. 21870076) with 10% FBS (Biological Industries, cat. no. 04-001-1A) to a final volume of 2 ml followed by incubation for 2 h at 37 °C with collagenase (2 mg ml^−1^, Sigma-Aldrich, cat. no. C5138) and DNase (0.03 mg ml^−1^, Sigma-Aldrich, cat. no. DN25) on a MACS vortex mixer. The reaction was halted by addition of fresh medium and the cell suspension was filtered through a 70-μm cell strainer (Corning, cat. no. CLS431751). The 15-ml suspension was centrifuged at 500*g* for 10 min, after which the pelleted cells were resuspended, counted, stained and analyzed by flow cytometry.

### Bone marrow-derived macrophages

Following euthanasia, mice were immersed in 75% alcohol for 10 min. Subsequently, bilateral hind limbs were excised and immersed in conical tubes containing 5% FBS and 5% penicillin–streptomycin (Sigma-Aldrich, cat. no. V900929) in Dulbecco’s Modified Eagle Medium (DMEM; Biological Industries, cat. no. 01-052-1ACS). A 1.0-ml syringe was employed to instill DMEM containing 10% FBS and penicillin–streptomycin into the bone marrow cavity until the backbone appeared white. The bone marrow washing solution was then transferred to a 15.0-ml centrifuge tube, and a volume 5× that of the red blood cell lysate was added. After coincubation for 10 min, the cells were centrifuged at 2,000 rpm for 5 min. The supernatant was discarded, and the cells were resuspended in DMEM containing 10% FBS, 1% penicillin–streptomycin and 50 ng ml^−1^ mouse macrophage colony-stimulating factor (Peprotech, cat. no. 315-02). The cells were cultured in a cell culture dish for 4 days. The original medium was aspirated, and fresh DMEM containing mouse macrophage colony-stimulating factor was added. After an additional 3 days of culture, the medium was removed, and the cells were washed three times with sterile phosphate-buffered saline (PBS) to eliminate nonadherent cells. The bone marrow-derived macrophages were gently scraped using a cell scraper, and a single-cell suspension was prepared and quantified for subsequent experiments.

### Construction and characterization of Man/LNP@piENOX2 INH

Ultrapure water was used to prepare 50 ml solutions of 100 mM citric acid (molecular weight of 210.14 g mol^−1^, weighing 1.05 g) and sodium citrate (molecular weight of 294.10 g mol^−1^, weighing 1.47 g). A volume of 33.0 ml of the citric acid solution was combined with 17.0 ml of the sodium citrate solution and mixed thoroughly, and diethylpyrocarbonate (DEPC) was added and allowed to stand for 30 min and subsequently autoclave to eliminate DEPC. Post sterilization, the volume was adjusted to 100 ml using DEPC-treated water, resulting in a 50 mM citrate buffer with a pH of 4.

The piENOX2 inhibitor (piENOX2 INH) was diluted to a concentration of 0.087 mg ml^−1^ in DEPC-treated 50 mM sodium citrate buffer, pH 4. An 8 mM phospholipid-ethanol solution was prepared according to the ratio SM-102:DMG-PEG2000:DSPC:cholesterol of 50:1.5:10:38.5. Within a microfluidic device, the phospholipid-ethanol solution and piENOX2 INH-citrate buffer were mixed at a volume ratio of 1:3 (9.575:28.725 ml) and a flow rate of 0.8:2.4 ml min^−1^. The effluent was collected after stabilization of the flow rate. Following sample collection, the ethanol concentration was diluted to less than 1% using 30× the volume of PBS. The solution was passed through a Millipore 30KD ultrafiltration tube, with centrifugation at 4,000 rpm for 15 min for ultrafiltration, and a small aliquot of the ultrafiltered solution was removed for particle size analysis.

A 0.9 mg sample of DSPE-PEG-Man (1% molar ratio) was dissolved in a small quantity of ethanol and added it to the ultrafiltered solution with vigorous vortexing. PBS was added for subsequent ultrafiltration to eliminate residual ethanol, adjusting the volume of PBS to 11 ml, and a small portion was removed for the assessment of encapsulation efficiency and particle size.

### Small animal imaging in vivo

The CIA mouse model was successfully established following the aforementioned procedural guidelines. On the 30th day subsequent to the initial immunization, a therapeutic dosage of Indocyanine Green (ICG)-labeled Man/LNP@piENOX2 INH was administered *via* the tail vein. The fluorescence distribution was monitored at predetermined intervals using a small animal in vivo imaging system to assess the targeted localization of Man/LNP@piENOX2 INH in the CIA mice.

### Man/LNP@piENOX2 INH in vivo treatment

Following establishment of the CIA mouse model, treatment with 2 mg kg^−1^ of the of piENOX2 inhibitor. The control mice received an equivalent dose of Man/LNP@piNC INH. Treatments were given every 3 days, for a total of six. Measurement of hind-paw thicknesses and arthritis scores were conducted at 3-day intervals. The mice were killed on day 54 following the first immunization, and serum, inguinal lymph node and bilateral hind-limb samples were collected.

### meRIP-seq

The meRIP-seq service was provided by Guangzhou RiboBio. Briefly, the procedure used Qubit and Agilent2200 to evaluate antibodies that selectively enrich m^6^A-modified RNA fragments. Following quality assessment of the samples, library construction was undertaken. A Illumina sequencing platform in PE150 mode was used for high-throughput sequencing. The data were analyzed in accordance with the meRIP bioinformatics pipeline.

The bioinformatics analysis involved multiple stages, including data quality control, sequence alignment to the genome, detection of binding peaks (Peak), prediction and annotation of motifs, annotation of peaks and associated statistical analyses. Further analyses involve the identification of differential peaks, as well as KEGG and GO analyses of genes associated with the peaks.

### meRIP–qPCR

The findings from meRIP-seq and the SRAMP database (https://www.cuilab.cn/sramp) were integrated to predict the putative m^6^A site within the *Itga4* gene. Subsequently, meRIP–qPCR primers were designed and synthesized by Guangzhou RiboBio. Following the respective treatment of RAW 264.7 cells with piENOX2 mimics and inhibitors, total RNA (in excess of 100 μg) was collected at predefined time points. meRIP–qPCR was performed in accordance with the instructions provided by the riboMeRIP m^6^A Transcriptome Profiling Kit (RiboBio, cat. no. R11096.6).

### Statistics

All experiments were performed at least three times. All data are presented as means ± standard deviation and were analyzed using GraphPad Prism 8.0. The differences between the two groups were analyzed using *t*-tests. For comparisons between more than two groups, one-way analysis of variance and Tukey’s or Dunnett’s multiple comparison tests were used. *P* < 0.05 was considered statistically significant.

## Results

### Macrophage-derived piENOX2 plays an important role in the onset and progression of RA

**Fig. 1:**
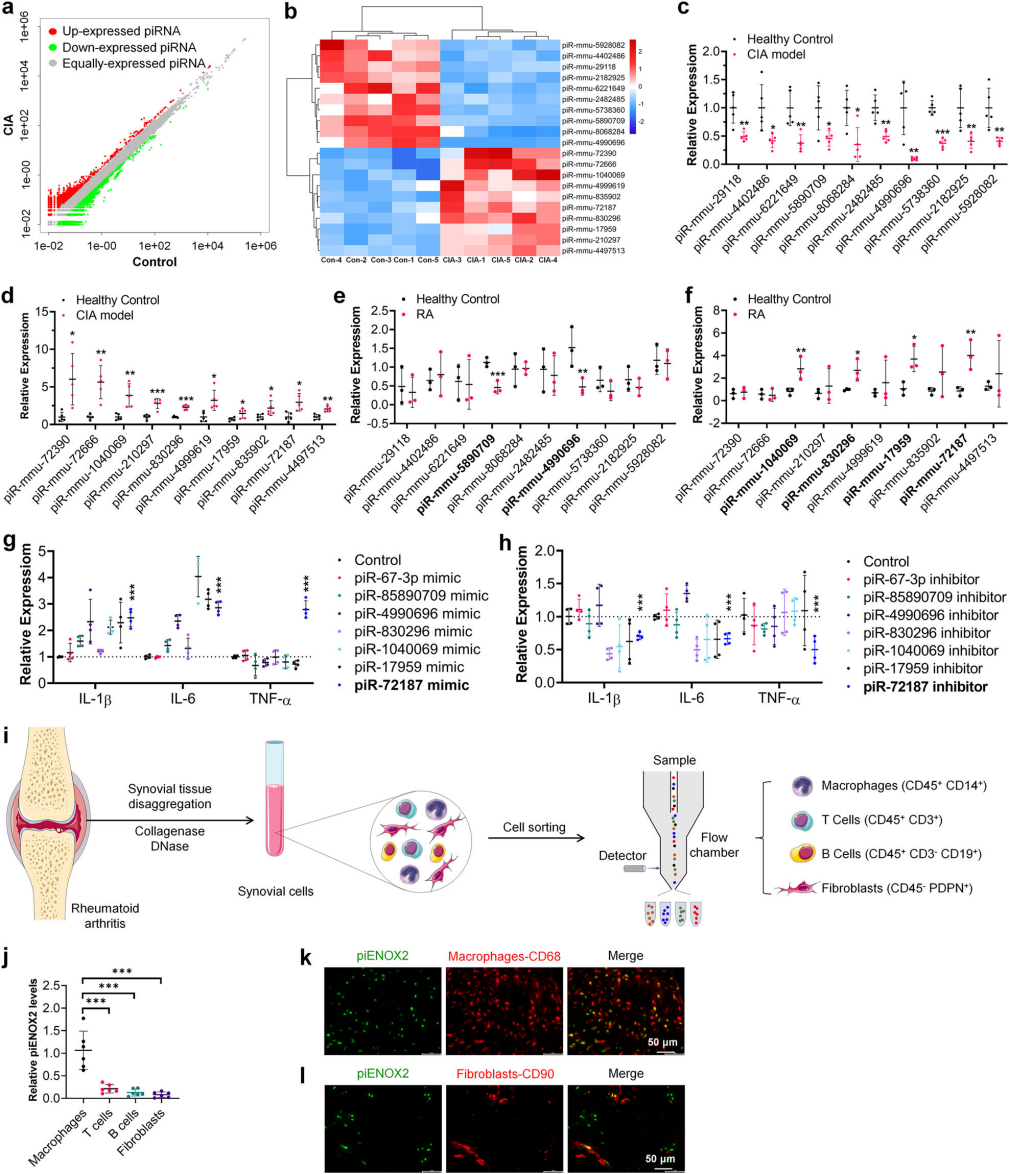
Macrophage-derived piENOX2 plays an important role in the progression of RA. **a** The piRNA expression in each sample was analyzed using the Illumina HiSeq 2500 system (*N* = *5*). The CIA mouse model was constructed, and hind-limb knee joint synovial tissue samples were collected at predetermined time points for total RNA extraction. **b**–**d** A cluster analysis and expression profiling of the top ten human–mice homologous piRNAs with the most significant upregulation and downregulation. **e**, **f** RT–qPCR was used to detect the expression of these piRNAs in the synovial tissue of patients with RA; in the LPS-induced inflammation model, piRNA mimics (**g**) and inhibitors (**h**) were used to overexpress and inhibit the expression of corresponding piRNAs, respectively, and RT–qPCR was performed to measure the levels of TNF, IL-1β and IL-6. The synovial tissue samples from the affected joints of CIA mice were isolated, and single-cell suspensions were prepared through enzymatic hydrolysis. **i**, The key cell populations in the synovial tissue, including synovial macrophages (CD45^+^CD14^+^), T cells (CD45^+^CD3^+^), B cells (CD45^+^CD3^−^CD19^+^) and fibroblasts (CD45^−^PDPN^+^), were sorted and collected via flow cytometry. **j**, RT–qPCR analysis of piENOX2 expression in individual cell populations (*N* = *6*). **k** The distribution and colocalization of piENOX2 and synovial macrophages (CD68^+^) in the synovial tissue of patients with RA were observed using FISH. **l** The distribution and colocalization of piENOX2 and fibroblasts (CD90^+^) in the synovial tissue of patients with RA were observed using FISH. * *P*< 0.05, ** *P*< 0.01, *** *P*< 0.001.

In the CIA model established in DBA1/J mice, synovial tissue piRNA expression was compared between diseased mice and healthy controls (Fig. 1a). The top ten most significantly upregulated or downregulated piRNAs were identified through comparative analysis of human and mouse sequences (Fig. 1b–d). RT–qPCR confirmed the expression trends of six piRNAs in the synovial tissues of patients with RA (Fig. 1e, f). Mimics and inhibitors for these piRNAs were synthesized, and their impact on proinflammatory cytokine expression (IL-1β, IL-6 and TNF) was verified in an LPS-induced RAW 264.7 cell inflammatory model (Fig. 1g, h). Notably, the piRNA-72187 mimic significantly increased the levels of proinflammatory cytokines, while the piRNA-72187 inhibitor suppressed these levels.

piRNA-72187, which originates from the *Enox2* gene, was designated as piENOX2. Using RNA pulldown, proteins binding to piENOX2 were enriched and isolated. Camas brilliant blue staining revealed a distinct band at 98 kD (Supplementary Fig. 1a), while electrophoresis confirmed the presence of PIWI-1 and PIWI-4 among the enriched proteins (Supplementary Fig. 1b), verified by protein profiling (Supplementary Fig. 1c–e). RNA coimmunoprecipitation–qPCR confirmed the presence of piENOX2 expression in the RNA bound to PIWI-1 and PIWI-4 (Supplementary Fig. 1f). Molecular docking simulations confirmed piENOX2 binding to PIWI-1 and PIWI-4 (Supplementary Fig. 1g,h), verifying the identity of the small RNA as piRNA.

Flow cytometry and FISH identified macrophages as the primary source of piENOX2 in synovial tissue. Flow cytometry was used to sort fibroblasts (CD45^−^PDPN^+^), macrophages (CD45^+^CD14^+^), T cells (CD45^+^CD3^+^) and B cells (CD45^+^CD3^−^CD19^+^) (Fig. 1i and Supplementary Fig. 2a). The RT–qPCR results indicated significantly higher piENOX2 expression in macrophages (Fig. 1j). FISH analysis further demonstrated a pronounced colocalization of piENOX2 with macrophages (CD68^+^) (Fig. 1k). Conversely, the expression of piENOX2 was minimal in fibroblasts (CD90^+^) without significant colocalization (Fig. 1l). These results suggest that macrophages are the main source cells of piENOX2.

**Fig. 2:**
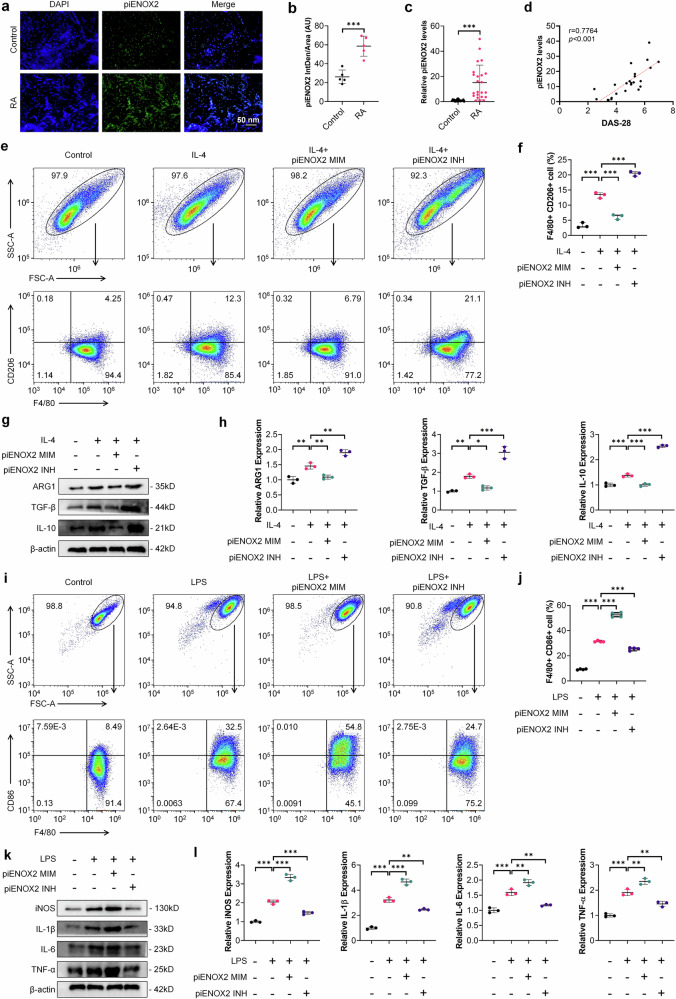
piENOX2 is upregulated in the synovial tissue of patients with RA and is involved in the regulation of macrophage polarization. **a** FISH observation of piENOX2 expression and distribution in the synovial tissue of patients with RA and control patients. **b** Semiquantitative analysis of piENOX2 fluorescence intensity in different samples using ImageJ (arbitrary units (AU)). **c** RT–qPCR detection of piENOX2 expression in the synovial tissue of patients with RA and controls (*N*_control_ = 20, *N*_RA_ = *23*). **d** A Pearson correlation analysis was performed to evaluate the association between DAS-28 scores and piENOX2 expression levels (*N* = 24). **e**, **f** Flow cytometry (**e**) and quantitative analysis (**f**) of the regulatory effects of piENOX2 MIM and piENOX2 INH on IL-4-induced M2 polarization (*N* = 3). **g**, **h** Western blotting (**g**) showing the regulatory effects of piENOX2 MIM and piENOX2 INH on the expression of M2 macrophage markers and their secreted cytokines, including ARG1, TGF-β and IL-10, with semiquantitative analysis (**h**) performed using ImageJ (*N* = 3). **i**, **j** Flow cytometry (**i**) and quantitative analysis (**j**) of the regulatory effects of piENOX2 MIM and piENOX2 INH on LPS-induced M1 polarization (*N* = 4). **k**, **l** Western blot analysis (**k**) of the regulatory effects of piENOX2 MIM and piENOX2 INH on the expression of M1 macrophage markers and their secreted cytokines, including iNOS, IL-1β, IL-6 and TNF, with semiquantitative analysis (**l**) performed using ImageJ (*N* = 3). *P < 0.05, **P < 0.01, ***P < 0.001.

Synovial tissue samples from patients with RA and controls were analyzed (Supplementary Fig. 2b,c). Hematoxylin and eosin staining revealed hyperplasia of synovial tissue in patients with RA (Supplementary Fig. 2d). The immunohistochemistry (IHC) and Immunofluorescence (IF) results showed elevated levels of the proinflammatory cytokines IL-1β, IL-6 and TNF in RA synovial tissue (Supplementary Fig. 2e–h), while FISH indicated increased piENOX2 expression in RA synovial tissue (Fig. 2a, b). RT–qPCR measurements confirmed the presence of significantly higher piENOX2 expression in RA synovial tissue (Fig. 2c). The DAS-28 was employed to assess the disease activity of patients with RA. Pearson correlation analysis revealed a significant positive association between DAS-28 scores and the expression level of piENOX2 (Fig. 2d). Flow cytometry analysis showed that the piENOX2 mimic (piENOX2 MIM) inhibited macrophage M2 polarization, decreasing F4/80^+^CD206^+^ cells, while the piENOX2 inhibitor (piENOX2 INH) facilitated M2 polarization (Fig. 2e, f). Western blotting of M2 macrophage markers (ARG1, TGF-β and IL-10) showed that piENOX2 MIM suppressed and piENOX2 INH enhanced their protein expression (Fig. 2g, h). In the LPS-induced macrophage M1 polarization model, piENOX2 MIM promoted M1 polarization, increasing the number of F4/80^+^CD86^+^ cells, while this was suppressed by piENOX2 INH (Fig. 2i, j). Western blotting confirmed that piENOX2 MIM upregulated and piENOX2 INH downregulated M1 macrophage markers (iNOS, IL-1β, IL-6 and TNF) (Fig. 2k,l).

These findings suggest that macrophage-derived piENOX2 promotes macrophage M1 polarization and upregulates proinflammatory cytokines, while its inhibition promotes macrophage M2 polarization, inhibiting inflammation.

### Macrophage-targeted piENOX2 INH liposome prodrug (Man/LNP@piENOX2 INH) effectively alleviates disease progression in CIA mice

**Fig. 3:**
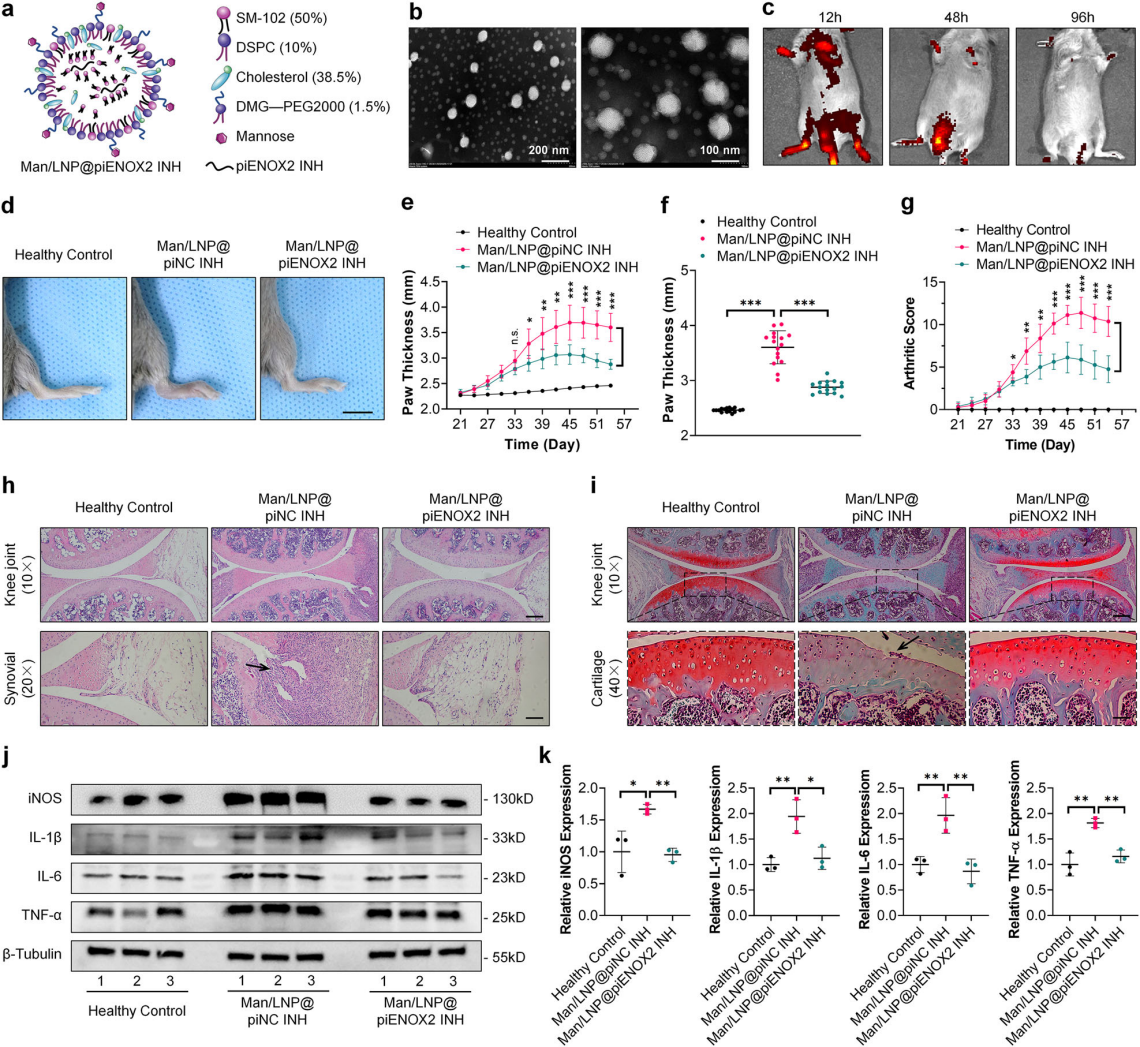
Man/LNP@piENOX2 INH effectively alleviates disease progression in CIA mice. **a** The construction of Man/LNP@piENOX2 INH. **b** TEM image of Man/LNP@piENOX2 INH. **c** The tissue distribution of ICG-labeled Man/LNP@piENOX2 INH in CIA mice, observed via small animal in vivo imaging at predetermined time points following tail vein injection. **d** Photographs of hind paws in each group of mice; scale bar, 10 mm. **e** Trend of changes in hind-paw thickness over the treatment period. **f** Final measured hind-paw thickness in each group. **g** Trends in arthritis scores of mice in each group throughout the treatment period. **h** A hematoxylin and eosin staining showing joint morphology and synovial infiltration in each group of mice; scale bars, 200 μm at 10× and 100 μm at 20× magnification. **i** Safranin-O and Fast Green staining indicating joint cartilage damage in each group of mice; scale bars, 200 μm at 10× and 50 μm at 40× magnification. **j**, **k** The protein expression levels of iNOS, IL-1β, IL-6 and TNF in the synovial tissue of each group of mice, detected by western blotting (**j**), with semiquantification (**k**) using ImageJ (*N* = 3). *P < 0.05, **P < 0.01, ***P < 0.001.

To verify the in vivo therapeutic efficacy of piENOX2 INH in CIA mice, we used liposomes as the drug carrier for piENOX2 INH, employing mannose (Man) for macrophage-targeted delivery^21^. Lipid nanoparticles (LNPs) are known to be superior carriers for small RNA drugs^22^. The synthesized LNPs were composed of SM-102 (50%), DSPC (10%), cholesterol (38.5%) and DMG-PEG_2000_ (1.5%) (Fig. 3a). The overall encapsulation efficiency for piENOX2 INH was 90.6%, with a concentration of 1323 ng μl^−1^. Characterization of the intermediate and final products indicated a hydrated particle size of LNP@piENOX2 INH of 99.39 nm, with a polymer dispersity index of 0.05744 and a zeta potential of 8.747 mV (Supplementary Fig. 3a,b). The hydrated particle size of Man/LNP@piENOX2 INH was approximately 114.3 nm, with a polymer dispersity index of 0.1022 and a zeta potential of −1.139 mV (Supplementary Fig. 3c,d). Transmission electron microscopy confirmed the spherical shape of Man/LNP@piENOX2 INH, with a particle size of about 97.7 nm (Fig. 3b). The targeting ability of ICG–Man/LNP@piENOX2 INH was verified using a small animal in vivo imaging system. Strong fluorescence signals were observed in the joints and claws of mouse limbs after 12 h, which were reduced 48 h while remaining detectable. By 96 h, the fluorescence signal had weakened significantly and nearly disappeared, indicating effective targeting and accumulation of Man/LNP@piENOX2 INH in the affected joint areas (Fig. 3c).

We systematically verified the therapeutic efficacy of Man/LNP@piENOX2 INH in CIA mice (Supplementary Fig. 3e). Man/LNP@piENOX2 INH did not induce structural or functional changes in various organs (Supplementary Fig. 3f). Analysis of paw swelling revealed significant reductions throughout the treatment cycle for mice in the Man/LNP@piENOX2 INH group, while the control group exhibited substantial swelling (Fig. 3d, f). Arthritis scores indicated effective alleviation of inflammatory symptoms in the Man/LNP@piENOX2 INH group compared with the controls (Fig. 3g).

Histological evaluations (hematoxylin and eosin, Safranin-O and Fast Green) demonstrated that Man/LNP@piENOX2 INH effectively inhibited synovial tissue proliferation and infiltration in the affected joints (Fig. 3h and Supplementary Fig. 3g). The loss of proteoglycans in the affected cartilage was alleviated in the treatment group, with maintenance of an intact articular surface and protection of chondrocyte counts and cartilage thickness (Fig. 3i and Supplementary Fig. 3h). The IHC results showed that Man/LNP@piENOX2 INH inhibited catabolism and enhanced anabolism in cartilage (Supplementary Fig. 3i). Micro-Computed Tomography (Micro-CT) data indicated that Man/LNP@piENOX2 INH effectively mitigated bone loss in affected joints (Supplementary Fig. 3j).

Flow cytometry was used to evaluate the impact of Man/LNP@piENOX2 INH on immune regulation. CIA mice showed elevated CD4^+^ T cell numbers, with reduced proportions of CD8^+^ T cells, increased IL-17A^+^ T cells (Th17 cells) and decreased Foxp3^+^ T cells (T_reg_). In the treatment group, the CD4^+^/CD8^+^ ratio was restored to normal levels^23^, accompanied by increased T_reg_ and reduced Th17 cells, reinstating immune homeostasis (Supplementary Fig. 4a,b). Enzyme linked immunosorbent assay (ELISA) results indicated a significant reduction in the serum levels of proinflammatory cytokine in the treatment group (Supplementary Fig. 4c). Although IL-10 levels in CIA mice were not significantly different from those in healthy controls, the treatment group showed significantly increased IL-10 concentrations. Western blotting showed that Man/LNP@piENOX2 INH inhibited IL-1β, IL-6 and TNF expression in synovial tissue (Fig. 3j, k). IHC results conformed these findings, showing reduced expression of these cytokines in articular cartilage and synovial tissue (Supplementary Fig. 4d).

In summary, Man/LNP@piENOX2 INH was observed to protect the structural integrity of articular cartilage, while maintaining immune homeostasis, suppressing inflammation and slowing disease progression in CIA mice.

### piENOX2 regulates macrophage polarization by targeting *Alkbh5* mRNA

**Fig. 4:**
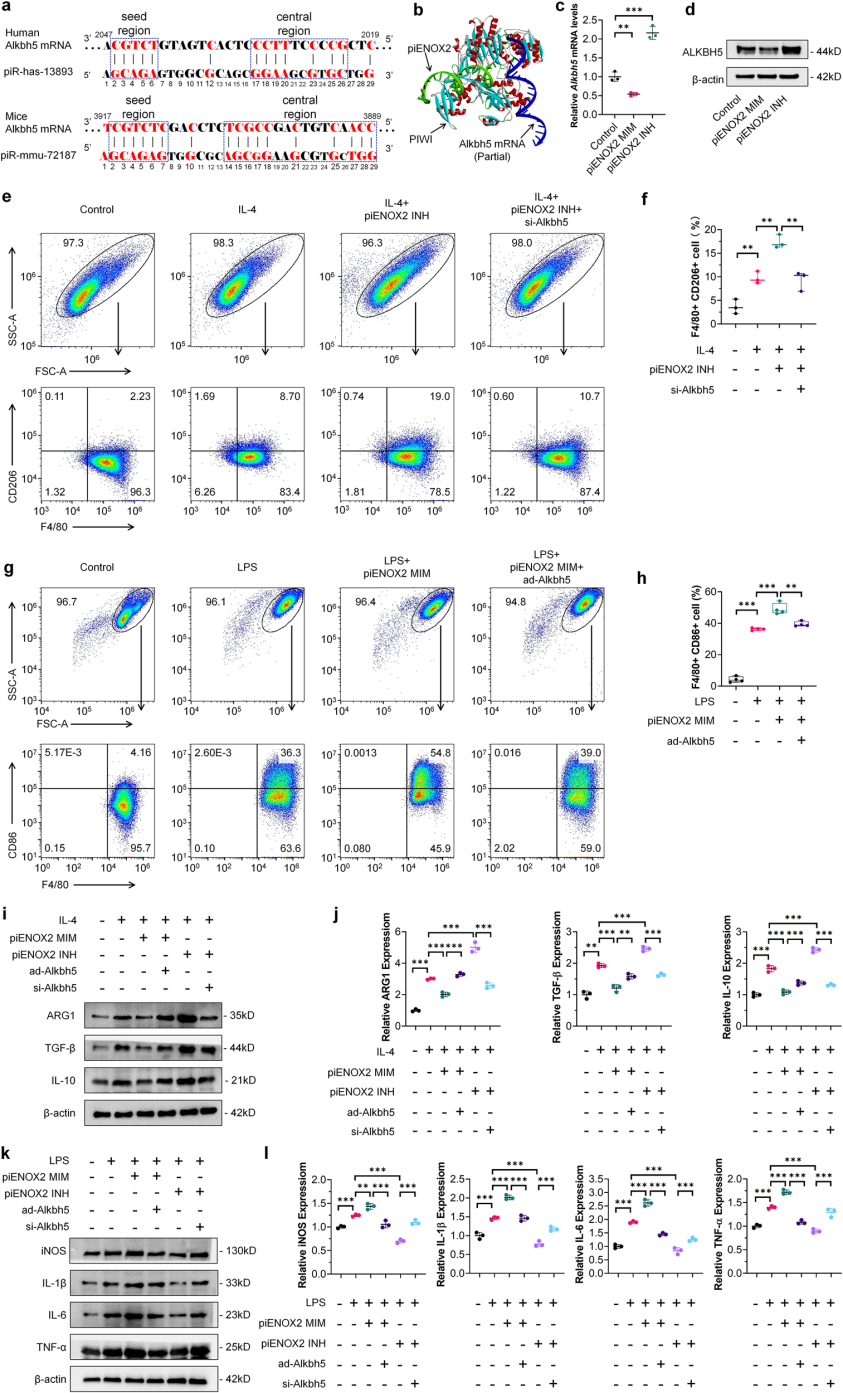
piENOX2 regulates macrophage polarization by targeting the degradation of *Alkbh5* mRNA. **a** Base complementarity between piENOX2 (human piRNA: piR-has-13893) and *Alkbh5* mRNA in humans and mice. **b** A simulation of the binding between piENOX2, PIWI and *Alkbh5* mRNA using visual molecular docking. **c**, **d** RT–qPCR (**c**) and western blots (**d**) showing the effects of piENOX2 MIM and piENOX2 INH on *Alkbh5* mRNA and protein expression (*N* = 3). **e**, **f** Flow cytometry (**e**) and quantitative analysis (**f**) of the effect of *Alkbh5* knockdown on piENOX2 INH-mediated promotion of M2 macrophage polarization (*N* = 3). **g**, **h** Flow cytometry (**g**) and quantitative analysis (**h**) of the effect of *Alkbh5* overexpression on piENOX2 MIM-mediated promotion of M1 macrophage polarization (*N* = 4). **i**, **j** The evaluation of the effect of *Alkbh5* overexpression or knockdown on piENOX2-regulated M2 macrophage polarization by western blot (**i**), with semiquantification (**j**) using ImageJ (*N* = 3). **k**, **l** The evaluation of the effect of *Alkbh5* overexpression or knockdown on piENOX2-regulated M1 macrophage polarization by western blotting (**k**), with semiquantification (**l**) using ImageJ (*N* = 3). *P < 0.05, **P< 0.01, ***P< 0.001.

Recent investigations have highlighted the regulatory functions of the piRNA/PIWI complex in modulating the expression of protein-coding genes through a mechanism involving complementary base pairing recognition^24,25^. At the molecular level, piRNA shares similarities with miRNA, functioning as a guide for AGO and PIWI proteins to target and cleave specific mRNA. To delve into the mechanistic intricacies of piENOX2, we utilized piRNA sequences, together with BLAST comparisons and screening using the piRBase and NCBI databases. Notably, we identified a piENOX2 recognition region on *Alkbh5* mRNA (Fig. 4a). Molecular docking was used to investigate the binding sites among piENOX2, PIWI protein and *Alkbh5* mRNA (Fig. 4b). We evaluated the regulatory impact of piENOX2 on *Alkbh5* mRNA and protein expression using RT–qPCR and western blotting. The results showed that the piENOX2 MIM reduced *Alkbh5* mRNA and protein levels, while the piENOX2 INH increased their expression (Fig. 4c, d). Further investigation in inflammatory cell models and the synovial tissue of CIA mice revealed significantly lower *Alkbh5* mRNA levels in the LPS-induced RAW 264.7 inflammatory cells, while *Alkbh5* mRNA levels were reduced in CIA mouse synovial tissue (Supplementary Fi. 5a,b). This suggests that heightened piENOX2 expression in the synovial tissue of inflammatory cell models and CIA mice may lead to targeted inhibition of *Alkbh5* mRNA and protein expression.

Subsequent exploration investigated the role of ALKBH5 in piENOX2-mediated regulation of macrophage polarization by overexpressing or knocking down ALKBH5 expression using an *Alkbh5* overexpression adenovirus (ad-Alkbh5) and siRNA (si-Alkbh5) (Supplementary Fig. 5c,d). The flow cytometry results showed that knockdown of *Alkbh5* inhibited the promacrophage M2 polarization effect of piENOX2 INH (Fig. 4e, f), while overexpression of *Alkbh5* blocked the ability of piENOX2 MIM to promote macrophage M1 polarization (Fig. 4g, h). The western blotting results further showed that overexpression of *Alkbh5* restored the expression of the M2 macrophage-related markers ARG1, TGF-β and IL-10 inhibited by piENOX2 MIM, while knockdown of *Alkbh5* blocked the promoting effect of piENOX2 INH on the expression of M2 macrophage-related markers (Fig. 4i,j). By contrast, in the LPS-induced macrophage M1 polarization model, overexpression of *Alkbh5* reduced the stimulatory effect of piENOX2 MIM on the expression of proinflammatory cytokines such as iNOS, IL-1β, IL-6 and TNF (Fig. 4k,l). Similarly, knockdown of *Alkbh5* blocked the inhibitory effects of piENOX2 INH on the expression of proinflammatory cytokines.

Collectively, these findings suggest that piENOX2/PIWI complex inhibits the expression of *Alkbh5* mRNA through targeted degradation, thereby inducing M1 polarization and activation of macrophages.

### The therapeutic efficacy of Man/LNP@piENOX2 INH is reduced in CIA model mice with myeloid-cell-specific-knockout of *Alkbh5*

**Fig. 5:**
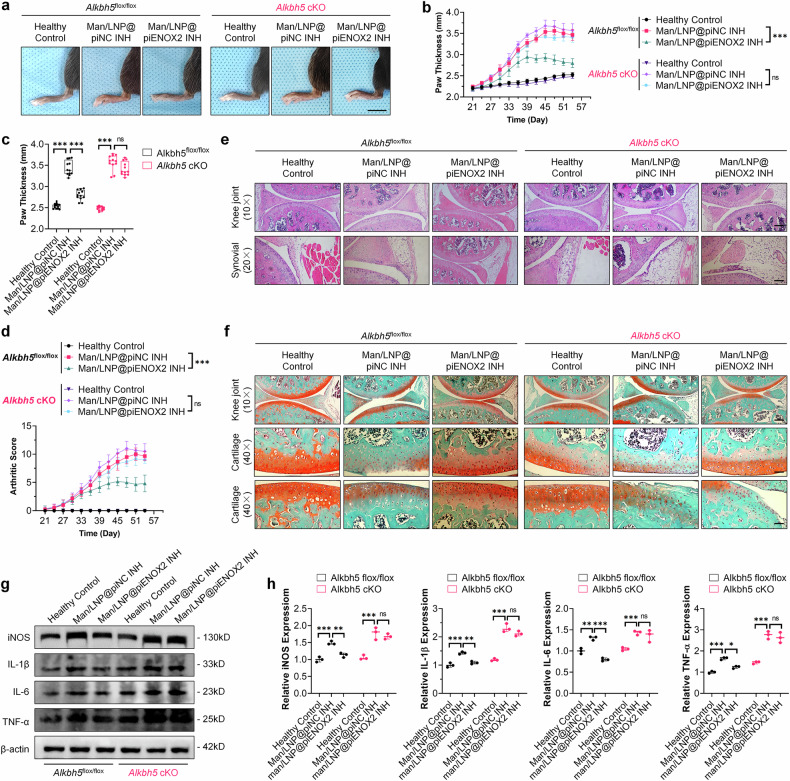
Knockout of *Alkbh5* blocks the effect of Man/LNP@piENOX2 INH on delaying disease progression in CIA mice. **a** An observation of swelling and redness in the hind paws of CIA mice in each treatment group at predetermined time points following treatment with different drugs; scale bar, 10 mm. **b**, **c** The trend of hind-paw thickness changes over the entire treatment period (**b**) and final measurement of hind-paw thickness in each group (**c**) (*N* = 6). **d** The trends in arthritis scores of mice in each group throughout the treatment period (*N* = 6). **e** Hematoxylin and eosin staining showing joint morphology and synovial infiltration in each group of mice; scale bars, 200 μm at 10× and 100 μm at 20× magnification. **f** Safranin-O and Fast Green staining indicating joint cartilage damage in each group of mice; scale bars 200 μm at 10× and 50 μm at 40× magnification. **g**, **h** Protein expression levels of iNOS, IL-1β, IL-6 and TNF in the synovial tissue of each group of mice, detected by western blotting (**g**), with semiquantification (**h**) using ImageJ (*N* = 3). A statistical analysis was conducted on data from day 54. ns, no significance; *P < 0.05, **P < 0.01, ***P < 0.001.

To further confirm the involvement of ALKBH5 in piENOX2-mediated regulation of macrophage polarization and activation in mice, we established an *Alkbh5* myeloid-cell-specific-knockout system using the Cre-loxP conditional-knockout approach (Lyz2-cre; *Alkbh5*^flox/flox^, *Alkbh5* cKO) mice^26–28^ (Supplementary Fig. 5e–i). Subsequently, CIA mouse models were induced in both *Alkbh5* cKO mice and control *Alkbh5*^flox/flox^ mice, with each group undergoing drug treatment according to the established protocol. As depicted in Fig. 5a–d, within the control group featuring *Alkbh5*^flox/flox^ mouse models, Man/LNP@piENOX2 INH significantly reduced hind-paw swelling and lowered arthritis scores in CIA mice compared with Man/LNP@piNC INH, effectively retarding disease progression in CIA mice. However, in the *Alkbh5* cKO mouse model, the therapeutic effects of Man/LNP@piENOX2 INH resembled those of Man/LNP@piNC INH, with limited influence on the alleviation of hind-paw swelling and reducing arthritis scores (Fig. 5a–d). Moreover, the results of the histological analyses using hematoxylin and eosin and Safranin-O and Fast Green staining presented in Fig. 5e,f indicate that in the *Alkbh5*^flox/flox^ mouse model, Man/LNP@piENOX2 INH preserved the structural integrity and function of the joints, reduced proliferation and infiltration of synovial tissue, and mitigated damage to the cartilage. Conversely, in the *Alkbh5* cKO mouse model, the protective effects of Man/LNP@piENOX2 INH on reducing synovial tissue proliferation and preserving cartilage were decreased (Fig. 5e,f). The observations in the *Alkbh5* cKO group mirrored the severe synovial tissue infiltration and cartilage destruction observed in the Man/LNP@piNC INH treatment group.

Consistent patterns were evident in the modulation of immune homeostasis and inflammation. In the CIA model of *Alkbh5*^flox/flox^ mice, Man/LNP@piENOX2 INH demonstrated a noteworthy capacity to reduce excessive immune activation. This was manifested by the maintenance of the CD4^+^/CD8^+^ T lymphocyte ratio, downregulation of Th17 cell proportion and upregulation of the T_reg_ proportion (Supplementary Fig. 6a,b). By contrast, in the CIA model of *Alkbh5* cKO mice, the effects on maintenance of immune homeostasis and inhibition of inflammation maintenance and inflammation by Man/LNP@piENOX2 INH were markedly compromised. The proportions of CD4^+^/CD8^+^, Th17 and T_reg_ cells in the Man/LNP@piENOX2 INH treatment group closely resembled those observed in the Man/LNP@piNC INH treatment group, with no significant differences (Supplementary Fig. 6a,b). Importantly, Man/LNP@piENOX2 INH failed to lower the expression of proinflammatory cytokines, including iNOS, IL-1β, IL-6 and TNF, within the affected articular synovial tissue of the CIA model of *Alkbh5* cKO mice (Fig. 5g,h).

These findings indicate that the targeted knockout of *Alkbh5* in macrophages attenuates the chondroprotective and immune regulatory effects of Man/LNP@piENOX2 INH in CIA model mice. This confirms the inhibitory role of ALKBH5 in Man/LNP@piENOX2 INH, contributing significantly to slowing the progression of CIA in the mice.

### piENOX2 regulates ALKBH5-mediated m^6^A modification of *Itga4* mRNA, thereby influencing macrophage polarization through the PI3K–AKT signaling pathway

**Fig. 6:**
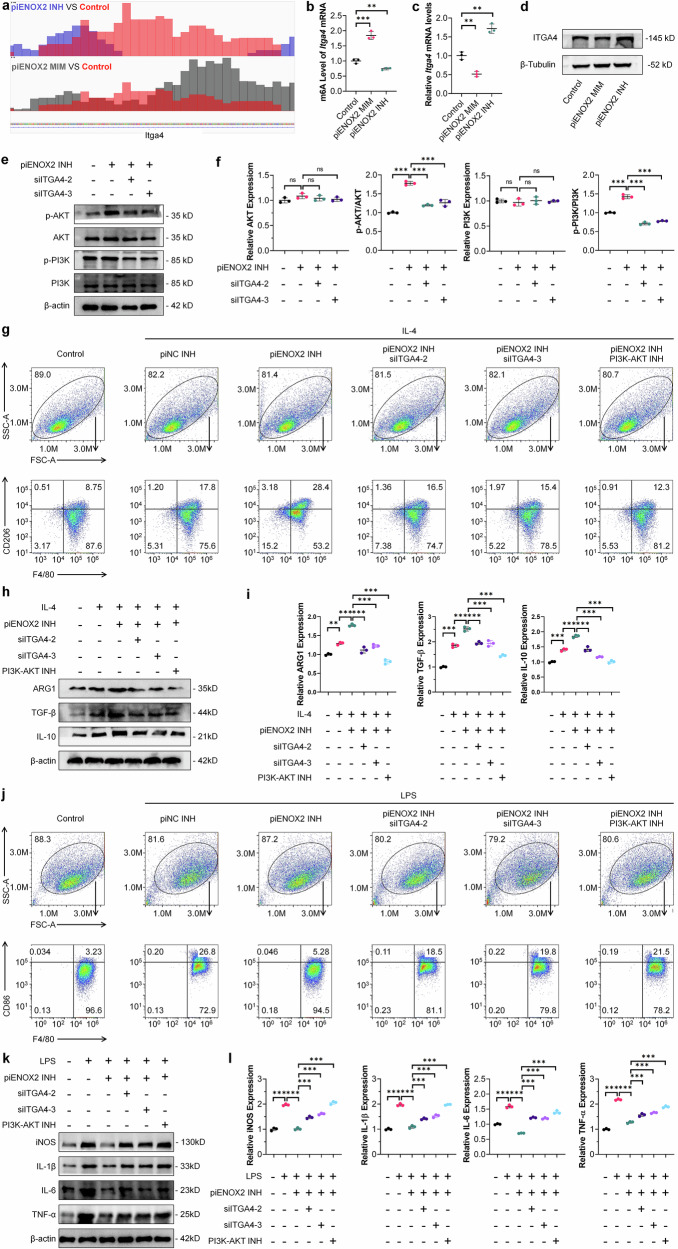
piENOX2 regulates ALKBH5-mediated m^6^A modification of *Itga4* mRNA, thereby influencing macrophage polarization through the PI3K–AKT signaling pathway. **a** An Integrated Genome Viewer visualization of the methylation level of *Itga4* mRNA. **b** meRIP–qPCR detection of *Itga4* m6A levels following treatment with piENOX2 MIM and piENOX2 INH (*N* = 3). **c** RT–qPCR detection of *Itga4* mRNA expression after treatment with piENOX2 MIM and piENOX2 INH (*N* = 3). **d** Western blotting of ITGA4 protein expression after treatment with piENOX2 MIM and piENOX2 INH. **e**, **f** Western blotting (**e**) showing the effects of the effects of *Itga4* knockdown on activation of the PI3K–AKT signaling pathway by piENOX2 INH, with semiquantification (**f**) using ImageJ (*N* = 3). **g** Flow cytometry analysis of the effects of *Itga4* knockdown or blocking the PI3K–AKT signaling pathway on piENOX2 INH-promoted M2 macrophage polarization (*N* = 4). **h**, **i** Western blotting (**h**) showing the effects of *Itga4* knockdown or blocking the PI3K–AKT signaling pathway on piENOX2 INH-promoted M2 macrophage polarization, with semiquantification (**i**) using ImageJ (*N* = 3). **j** Flow cytometry analysis of the effects of *Itga4* knockdown or blocking the PI3K–AKT signaling pathway on piENOX2 INH-inhibited M1 macrophage polarization (*N* = 4)*.* **k**, **l** Western blotting (**k**) showing the effects of *Itga4* knockdown or blocking the PI3K–AKT signaling pathway on piENOX2 INH-inhibited M1 macrophage polarization, with semiquantification (**l**) using ImageJ (*N* = 3). ns no significance; **P< 0.01, ***P < 0.001.

ALKBH5 is a demthylase involved in m^6^A modifications and plays a pivotal role in preserving the dynamic equilibrium of m^6^A modifications within the body^29–32^. Based on previous data, meRIP-seq was used to investigate changes in m^6^A levels during piENOX2 regulation of macrophage polarization. Treatment with piENOX2 MIM or piENOX2 INH had no significant effects on the m^6^A site region (Supplementary Fig. 7a). Since piENOX2 can inhibit ALKBH5 expression, we identified 26 genes with increased m^6^A levels in the piENOX2 mimic group and decreased m^6^A levels in the piENOX2 inhibitor group (Supplementary Fig. 7b). *Itga4*, among these genes, is known to be associated with Extracellular Matrix (ECM)-receptor interactions and the PI3K–AKT pathway. KEGG analysis showed enrichment in these pathways for both piENOX2 MIM versus control and piENOX2 INH versus control groups (Supplementary Fig. 7c,d). The Integrated Genome Viewer results showed that compared with the control group, *Itga4* mRNA had higher levels of m^6^A modification in the piENOX2 INH group and lower m^6^A levels in the piENOX2 MIM group. (Fig. 6a). meRIP–qPCR confirmed that piENOX2 MIM increased and piENOX2 INH decreased m^6^A levels of *Itga4* mRNA (Fig. 6b). In addition, meRIP–qPCR further confirmed that ALKBH5 expression levels significantly influence the m^6^A methylation status of *Itga4* mRNA (Supplementary Fig. 7e). Together, these findings support the notion that piENOX2 regulates the m^6^A modification of *Itga4* mRNA by modulating ALKBH5 expression levels. RT–qPCR and western blotting showed that piENOX2 MIM downregulates and piENOX2 INH upregulated ITGA4 mRNA and protein levels (Fig. 6c,d). meRIP-seq analysis indicated that ITGA4 regulated macrophage polarization by piENOX2 via the PI3K–AKT pathway. To explore this, we used two effective *Itga4* siRNAs (siITGA4-2 and siITGA4-3) (Supplementary Fig. 7f). In the LPS-induced model of M1 polarization, piENOX2 INH was found to activate the PI3K–AKT pathway, shown by increased phosphorylation of PI3K and AKT. Knockdown of *Itga4* reduced this phosphorylation and inhibited the pathway activation by piENOX2 INH (Fig. 6e,f).

To further understand the role of ITGA4, we blocked the pathway with siITGA4-2, siITGA4-3 and a PI3K–AKT inhibitor (PI3K–AKT INH). Flow cytometry showed piENOX2 INH promoted M2 polarization (Fig. 6g and Supplementary Fig. 7g). However, *Itga4* knockdown or treatment with the PI3K–AKT INH blocked this effect. Western blotting confirmed that siITGA4 and PI3K–AKT INH reduced M2 markers such as ARG1, TGF-β and IL-10 (Fig. 6h,i). In the M1 model, piENOX2 INH inhibited M1 polarization, but siITGA4-2, siITGA4-3 and PI3K–AKT INH weakened this inhibition (Fig. 6j and Supplementary Fig. 7h). Western blotting also showed that blocking the PI3K–AKT pathway affected piENOX2 INH suppression of M1 markers (iNOS, IL-1β, IL-6 and TNF) (Fig. 6k,l).

In summary, piENOX2 was found to regulate ALKBH5-mediated m^6^A modification of *Itga4* mRNA, which affects its mRNA stability and protein expression. This, in turn, influenced macrophage regulation through the PI3K–AKT signaling pathway.

## Discussion

The successful deployment of small nucleic acid drugs presents promising avenues for pioneering novel therapies in the context of RA. These include siRNA, antisense oligonucleotides and other small nucleic acid drugs, all of which have been found effective in clinical applications. piRNAs, discovered in 2006, represent a class of small noncoding RNA recognized for their substantial influence on the pathogenesis of diseases of the reproductive system, neurological disorders and tumors, among others^3–6^. Despite its acknowledged roles, piRNA has remained unexplored in the context of autoimmune disease treatment. In this investigation, we identified an piRNA derived from ENOX2, denoted as piENOX2, exhibiting increased expression in the synovial tissue of patients with RA and CIA mice, implicating its role in disease progression. In vitro assessments revealed that macrophage-derived piENOX2 promoted macrophage M1 polarization, leading to increased proinflammatory cytokine expression, while the inhibition of piENOX2 resulted in anti-inflammatory effects.

To evaluate the therapeutic efficacy of the piENOX2 inhibitor in CIA model mice, a liposome-based nanodrug delivery system was developed. Specifically modified with Man for targeted drug delivery to macrophages^21,33^, the liposome prodrug of the piENOX2 inhibitor, termed Man/LNP@piENOX2 INH, demonstrated targeted accumulation within the affected joint regions of CIA mice in in vivo observations. This formulation effectively inhibited the proliferation and infiltration of synovial tissue in the affected joints, preserving the structural integrity of cartilage tissue. Moreover, Man/LNP@piENOX2 INH was observed to modulate immune homeostasis, reducing the levels of systemic and local inflammatory cytokines within the joints. Consequently, this intervention significantly retarded disease progression in the CIA model mice. These findings collectively suggest that piENOX2 may represent a promising target for the treatment of RA.

Research has emphasized the functional similarities between piRNA and miRNA, acting as guides for AGO and PIWI proteins^34^. The piRNA/PIWI complex regulates protein-coding genes by cleaving mRNA through a complementary base-pairing mechanism. In 2018, Li et al. identified piRNA’s distinct targeting rules, emphasizing perfect base pairing in the 2–7 seed region^35^. A 2021 study by MacRae et al. revealed the piRNA/PIWI complex’s Cryogenic-electron microscopy structure, confirming and expanding on the recognition mechanism^25^. Structural differences between PIWI and AGO confer unique targeting properties to piRNA. The PIWI protein, requiring a shorter seed region, exhibits weaker binding than miRNA. However, piRNA compensates through its extended interaction with the central region, permitting a degree of mispairing. Notably, base pairing in the 3′ end is dispensable for target recognition. Following recognition, the PIWI protein acts as an endonuclease, cleaving target mRNA to modulate gene expression. In this study, the target mRNA of piENOX2, *Alkbh5*, was identified using sequencing data and the piRBase and NCBI databases. Overexpressed piENOX2 contributed to inflammation by targeting *Alkbh5* mRNA degradation in CIA mouse synovial tissue and cell models, affecting macrophage activation. Conversely, Alkbh5 mRNA knockdown impedes the anti-inflammatory effects of the piENOX2 inhibitor, highlighting the regulatory role of piENOX2 in macrophage polarization in RA progression.

ALKBH5, a demethylase associated with m^6^A modification, maintains dynamic equilibrium in physiological homeostasis^29–32^. RNA m^6^A modifications are dynamic and reversible and involve various enzymes and proteins, impacting pathophysiological processes. Cao et al. in 2019 showed that Mettl3-mediated m^6^A modification activates dendritic cells, influencing T cell activation^36^. He et al. demonstrated that YTHDF1-mediated messenger mRNA m^6^A modification’s role in antigen-specific immunity^37^. Li et al. found that Mettl3 deficiency decelerates Toll-like receptor-mediated macrophage activation, while IGF2BP2 deletion induces an augmented M1 phenotype, promoting colitis^38,39^. These studies underscore the involvement of m^6^A modifications in autoimmune disease progression by regulating macrophage activation and polarization.

**Fig. 7:**
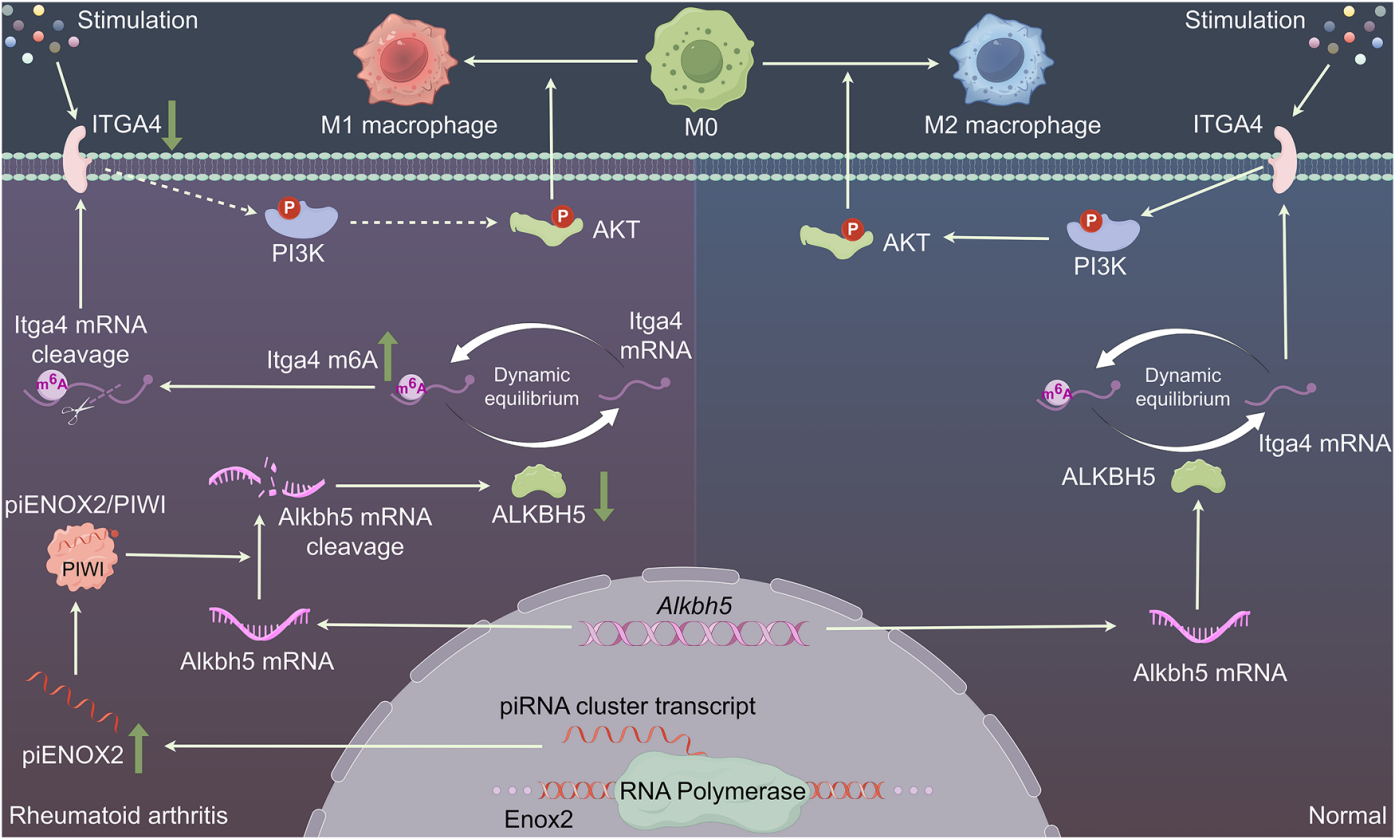
Schematic diagram of the mechanism by which piENOX2 accelerates RA disease progression. Under physiological conditions, ALKBH5 stabilizes *Itga4* mRNA expression through demethylation, subsequently activating the PI3K–AKT signaling pathway. This regulatory mechanism promotes macrophage M2 polarization, maintaining immune homeostasis. In RA, piENOX2 production is significantly elevated. piENOX2 selectively targets *Alkbh5* mRNA for degradation, thereby inhibiting ALKBH5 protein expression. This disruption leads to increased m^6^A modification on *Itga4*, reducing its mRNA stability and decreasing ITGA4 expression. As a result, ITGA4-mediated activation of the PI3K–AKT pathway is inhibited, promoting macrophage M1 polarization. This cascade of events ultimately contributes to the onset and progression of RA.

To elucidate the underlying mechanisms associated with piENOX2/ALKBH5 in RA progression, we conducted meRIP-seq and meRIP–qPCR analyses. The findings indicated that piENOX2 exerted a promoting effect on the m^6^A modification of *Itga4* mRNA, leading to a downregulation in its expression. This, in turn, suppressed activation of the PI3K–AKT signaling pathway. Indeed, a monoclonal antibody drug targeting ITGA4, natalizumab, is used for treating both multiple sclerosis and Crohn’s disease^40,41^. Despite the shared immunological nature of multiple sclerosis, Crohn’s disease and RA, Han et al. described a clinical case wherein the administration of natalizumab for multiple sclerosis resulted in the onset of RA^42^. Several studies have demonstrated a temporal association between natalizumab treatment and RA manifestation. This association is biologically plausible, and alleviation of RA symptoms was observed following the cessation of natalizumab treatment. This clinical observation underscores the distinctive immunopathogenesis between multiple sclerosis and RA. In the present study, we similarly observed that the downregulation of ITGA4 induced macrophage activation and M1 polarization, aligning with the findings from clinical cases. Furthermore, many earlier investigations have consistently demonstrated the involvement of the PI3K–AKT signaling pathway in the regulation of macrophage activation and polarization^43,44^. The results of the present study demonstrate that either knockdown of ITGA4 or pharmacological blocking the PI3K–AKT signaling pathway significantly impeded the promacrophage M2 polarization and the anti-inflammatory effects induced by the piENOX2 inhibitor. Based on the accumulated data, we posit that piENOX2 attenuates the expression of ALKBH5 protein by selectively targeting the degradation of *Alkbh5* mRNA. Consequently, this event leads to an elevation in the m^6^A modification level of *Itga4*, reducing mRNA stability and ITGA4 expression. This orchestrated molecular cascade, in turn, prevents ITGA4-mediated activation of the PI3K–AKT signaling pathway, ultimately promoting M1 polarization and macrophage activation, thereby driving the progression of RA, as illustrated in Fig. 7.

Effective modulation of synovial macrophage polarization is a promising strategy to slow RA progression. Recent research has demonstrated the complex role of piRNAs in disease pathogenesis. In patients with RA and CIA murine models, piENOX2 upregulation in synovial tissue was observed. Overexpressed piENOX2 stimulated M1 macrophage polarization. A liposomal prodrug of the piENOX2 inhibitor was effective in reducing synovial tissue proliferation and infiltration in CIA mice, preserving joint integrity. Mechanistically, the piENOX2 inhibitor stabilized ALKBH5 expression, reducing *Itga4* m^6^A modifications, sustaining PI3K–AKT pathway activation and thereby promoted macrophage M2 polarization and slowed the progression of RA. This study pioneers piRNA exploration in RA, offering epigenetic insights and potential for the development of precise therapeutics.

## Supplementary information

Supplementary Information

## Data Availability

All data generated for this study are available from the corresponding authors upon reasonable request.
