# Supplementary material for: piENOX2 regulates ALKBH5-mediated Itga4 m6A modification to accelerate the progression of rheumatoid arthritis
Source: Exp Mol Med. 2025 Jul 23;57(7):1579–92. doi: 10.1038/s12276-025-01503-3 (PMC12322066; doi:10.1038/s12276-025-01503-3)
Supplement: Supplementary file 1 — Supplementary Information [file 12276_2025_1503_MOESM1_ESM.pdf]

Supplementary materials

Materials and Methods

RNA pulldown

The biotin labeling of piENOX2 was performed by Guangzhou RiboBio Co., Ltd., concurrently with the construction of a negative control piRNA probe. The specific labeling protocol entailed a single phosphate modification at the 5' end of piENOX2 and a 2-methoxy modification at the 3' end of piENOX2. Following stimulation of RAW 264.7 cells with 1.0 μg/mL LPS, the total cellular protein was extracted, and RNA pulldown was performed as directed by the reagent manufacturer (Geneseed, Cat.P0202).

In summary, biotinylated piENOX2 and the control biotinylated piNC were separately incubated with cellular protein extracts to enable the complete interaction of piRNA with proteins. Magnetic beads were then used to selectively enrich proteins associating with piENOX2, and the bound proteins were eluted after thorough washing. Verification of the PIWI protein species binding to piENOX2 was achieved through either western blotting (utilizing Coomassie Brilliant Blue staining) or protein spectrometry analysis, the latter performed by Shanghai Bioprofile Co., Ltd.

RNA immunoprecipitation (RIP)

Following stimulation of RAW 264.7 cells with 1.0 μg/mL LPS, cell collection was performed at pre-determined time intervals, and RIP was performed as directed by the reagent manufacturer (Sigma−Aldrich, Cat.17-700). Briefly, cells were lysed to

form protein-RNA complexes, with separate incubation of PIWI-1 and PIWI-4 antibodies with the protein-RNA complexes. A/G magnetic beads were used for subsequent immunoprecipitation. The immunoprecipitated complexes were washed numerous times to eliminate nonspecifically bound proteins and other extraneous components. The immunoprecipitated complex underwent protease hydrolysis to segregate RNA and protein components. RNA extraction kits and additional methodologies were then applied to extract RNA from samples following enzymatic digestion of proteins. RT-qPCR was used to measure the expression levels of piENOX2 within RNA bound to PIWI-1 and PIWI-4.

The role of ALKBH5 in piENOX2 regulating macrophage activation and polarization

ALKBH5 small interfering RNA (si-Alkbh5) was designed and synthesized by Guangzhou RiboBio Co., Ltd., while an adenovirus for ALKBH5 overexpression (ad-Alkbh5) was constructed and synthesized by Hanbio Biotechnology Co. Ltd. In 12-well plates, RAW 264.7 cells were seeded and allowed to adhere before transfection with si-Alkbh5 and ad-Alkbh5 following established protocols. Subsequent to treatment with 1.0 μg/mL LPS or 20.0 ng/mL IL-4 (MCE, Cat.HY-P70445) for 48 hours, Western blotting was used to assess the expression levels of pro-inflammatory cytokines. Additionally, flow cytometry was utilized to quantify the proportions of F4/80$^+$CD86$^+$ macrophages and F4/80$^+$CD206$^+$ macrophages.

Therapeutic effect of Man/LNP@piENOX2 INH in *Alkbh5* cKO mice

Alkbh5$^{flox/-}$ mice and Lyz2-Cre mice were generated and bred by Cyagen Biosciences

(Guangzhou) Lnc. *Alkbh5* cKO mice and control *Alkbh5*$^{flox/flox}$ mice were bred as shown in Supplementary Fig. 5. Complete Freund's adjuvant was added to an equal volume of 2 mg/mL chick type II collagen (Chondrex, Cat.20012) on ice and emulsified using a homogenizer, taking care to prevent heat-induced denaturation. 10-week-old male *Alkbh5* cKO mice and control *Alkbh5*$^{flox/flox}$ mice were anesthetized with 0.1 mL of the emulsion was injected intradermally at two points. A booster with an emulsion of chick type II collagen in incomplete Freund's adjuvant was given on day 14.[1] Corresponding pharmaceutical agents were administered intravenously through the tail veins based on a standardized regimen. Mice from each cohort were euthanized at predetermined time intervals, and samples of blood, inguinal lymph nodes, and bilateral hind limbs were harvested for the assessment of joint preservation, immune modulation, and inflammation control efficacy within each experimental group.

Histopathological staining

At predetermined intervals, hindlimb specimens were obtained from mice in each experimental cohort. The soft tissues surrounding the knee joints were excised, fixed overnight in 4% (*w/v*) PBS-buffered paraformaldehyde (Beyotime, Cat.P0099), and subsequently subjected to decalcification in a 15% (*w/v*) EDTA solution (Solarbio, Cat.E1171) for 21 days. Following decalcification, the samples were washed, paraffin-embedded, and sectioned. Hematoxylin-Eosin staining (H&E, Beyotime, Cat.C0105M) and Safranin-O and Fast Green staining (Solarbio, Cat.G1371) were performed in accordance with the instructions provided by the respective reagent manufacturers. Microscopic examination and imaging were employed to evaluate joint morphology,

synovial infiltration, and cartilage preservation in the specimens from each experimental group.

Scoring assessments, including the Harris Hip Score (HSS) and Osteoarthritis Research Society International (OARSI) scores, were conducted on the samples from each group, adhering to the criteria outlined in Supplementary Table 1 and Supplementary Table 2, respectively.

Immunohistochemistry

Knee-joint specimens from mice were acquired as described, and underwent fixation, decalcification, embedding, and sectioning. Subsequently, sections from each experimental group underwent deparaffinization, antigen retrieval, and blocking of nonspecific binding as per the manufacturer's guidelines for the reagents employed (ZSGB-BIO, Cat.PV-8000). The sections were then incubated successively with primary and secondary antibodies. The expression and distribution of pro-inflammatory cytokines were assessed microscopically.

Quantification of T cell clustering within lymph nodes using flow cytometry

Mice from each experimental group were euthanized at predetermined intervals and then immersed in 75% ethanol for 5 minutes. A midline incision was made along the abdomen using scissors to fully expose the peritoneum. The groin skin was peeled off to reveal the transparent lymph nodes, measuring approximate gently 2.0-5.0 mm in diameter. Using forceps, the lymph nodes were gently isolated, placed in pre-cooled PBS, and transferred to a sterile work surface. A 6-well plate containing 1.5 mL of RPMI 1640 medium supplemented with 2% FBS was prepared. The lymph nodes were

placed in a 400-mesh strainer, and gentle grinding was performed using a 5.0 mL syringe plunger. Then, 0.5 mL of RPMI 1640 with 2% FBS was used to rinse the filter, with this step repeated twice. The resulting cell suspension was transferred to a 15.0 mL centrifuge tube. Additionally, 2.0 mL of RPMI 1640 containing 2% FBS was used to wash the 6-well plate, and the wash was combined with the cell suspension in the centrifuge tube. Centrifugation was performed at 1500 rpm for 5 minutes at 4°C, after which the supernatant was carefully removed. The pellet was resuspended in 1.0 mL of RPMI 1640 with 2% FBS. Following cell counting, flow cytometry staining detection was conducted.

Detection of macrophage polarization by flow cytometry

Following the procedure outlined for RAW 264.7 cell processing, cells were harvested and quantified at predetermined time points, then diluted to a concentration of $5*10^6$-$1*10^7$ cells/mL. Approximately 100 μL (corresponding to $5*10^5$-$1*10^6$ cells) was transferred into a 1.5 mL centrifuge tube, to which 150 μL of Cyto-Fast™ Fix/Perm Buffer (Biolegend, Cat.426803) was added. The mixture was mixed well and incubated at room temperature for 20 minutes. Following fixation, 1.0 mL of 1X Cyto-Fast™ Perm Wash Solution was added, and centrifugation was carried out at 350×g for 5 minutes, with subsequent removal of the supernatant. This washing procedure was repeated twice for thorough cleansing of the cells. Antibodies, prepared in 1 Cyto-Fast™ Perm Wash Solution, were then introduced into the experimental workflow. Specifically, 1.3 mL of 1X Cyto-Fast™ Perm Wash Solution was combined with 13.0 μL of F4/80 antibody, 26.0 μL of CD206 antibody, and 26 μL of CD86 antibody. One

hundred µl of this antibody mixture was added to each tube and incubated in the dark at room temperature for 20 minutes. Post-incubation, 1.0 mL of 1 × Cyto-Fast™ Perm Wash Solution was added, followed by centrifugation at 350×g for 5 minutes, and removal of the supernatant. This washing process was repeated to ensure proper antibody removal. The cells were then resuspended in 300 µL of PBS (containing 2% serum) per tube and analyzed by flow cytometry.

The flow cytometry antibodies were used: FITC Anti-mice CD3 (Biolegend, Cat.100203), PE/Cyanine7 anti-mice CD19 (Biolegend, Cat.103116), APC anti-mice CD14 (Biolegend, Cat.123311), APC/Cyanine7 Anti-mice CD45 (Biolegend, Cat.103116), Brilliant Violet 421™ Anti-mice CD25 (Biolegend, Cat.102033), APC Anti-mice CD4 (Biolegend, Cat.100412), Brilliant Violet 510™ Anti-mice CD8a (Biolegend, Cat.100751), PE Anti-mice FOXP3 (Biolegend, Cat.126403), Brilliant Violet 421™ Anti-mice IL-17A (Biolegend, Cat.506925), PE Rat IgG2b, κ Isotype Ctrl (Biolegend, Cat.400607).

PI3K/AKT signaling pathway

Itga4 siRNA was custom-designed and synthesized by Tsingke Biotechnology Co., Ltd. Subsequent to siRNA transfection of LPS-induced RAW 264.7 cell model followed a previously documented protocol, total protein samples were collected at predetermined time intervals. Western blotting was employed to assess ITGA expression, facilitating the identification of an optimal *Itga4* siRNA. Using the LPS-induced RAW 264.7 cell model, the effects of ITGA knockdown using Itga4 siRNA, as well as the blockade of the PI3K-AKT signaling pathway with a PI3K/AKT inhibitor, on the regulatory effects

of the piENOX2 inhibitor concerning macrophage activation and polarization were experimentally verified. The siRNA sequences are listed in Supplementary Table 3.

Real-Time Quantitative PCR (RT-qPCR)

Total RNA was extracted from cells or tissue samples using the HiScript II Q RT SuperMix (Vazyme, Cat.R223-01) for qPCR mini kit. The RNAs were then reverse-transcribed to cDNA, following the manufacturer's guidelines. Quantification of mRNA expression levels was conducted through RT-qPCR utilizing the ChamQ Universal SYBR qPCR Master Mix (Vazyme, Cat.Q711). The RT-qPCR primer sequences are listed in Supplementary Table 4.

Western blotting

Cells were chilled and washed with pre-cooled PBS, followed by lysis in ice-cold RIPA lysis buffer (Beyotime, Cat. P0013B) containing a cocktail of protease and phosphatase inhibitors (Solarbio, Cat. IP0280), with intermittent gentle agitation every 10 minutes over a 30-minute period. Clarification of cellular debris was achieved through centrifugation (15,000 rpm, 15 minutes, 4 °C). The supernatant was mixed with SDS sample buffer and denatured by boiling at 95 °C for 10 minutes. Proteins were separated on 8−12% Bis-Tris polyacrylamide gels using running buffer, transferred onto a polyvinylidene fluoride (PVDF, Millipore, Cat.IPVH00010) membrane, and blocked with 5% bovine serum albumin in PBS. Primary antibodies targeting the relevant proteins were incubated overnight at 4 °C. The PVDF membrane underwent three 10-minute washes with Tris-buffered saline-Tween (TBST). Subsequently, the corresponding secondary antibody was applied for 1 hour at room temperature. The

PVDF membrane underwent three additional 5-minute washes with TBST, and protein expression was visualized using a Chemiluminescence imaging system.

The protein extraction method for tissue samples differed slightly. First, an appropriate amount of tissue was weighed, placed in a mortar, and gently ground with the addition of liquid nitrogen. To prevent protein degradation, liquid nitrogen was added continuously during the grinding process until the tissue was transformed into a particle-free powder. Subsequently, 1.0 mL of RIPA lysis buffer, containing a cocktail of protease and phosphatase inhibitors, was added directly to the mortar. After equilibration to ambient temperature, the RIPA buffer was transferred to a 1.5 mL EP tube, underwent further lysis on ice for 30 minutes with intermittent gentle shaking every 10 minutes. The subsequent steps were the same as those used for protein extraction from cells.

The following primary antibodies were used: IL-1-beta Antibody (Proteintech, Cat. 16806-1-AP), IL-6 Antibody (Proteintech, Cat. 66146-1-lg and Cat.26404-1-AP); TNF-alpha Antibody (Proteintech, Cat.17590-1-AP), ALKBH5 Antibody (Abcam, Cat.ab195377), ITGA4 Antibody (Proteintech, Cat.194676-1-AP), PI3K Antibody (Affinity, Cat. AF8241), AKT Antibody (Proteintech, Cat. 10176-2-AP), p-PI3K Antibody (Affinity, Cat. AF3241), p-AKT Antibody (Proteintech, Cat. 66444-1-lg), PIWI1 Antibody (Proteintech, 15659-1-AP), PIWI2 Antibody (Proteintech, Cat.14209-1-AP), PIWI4 Antibody (Affinity, Cat.DF7207), Beta-Tubulin Antibody (Proteintech, Cat.10094-1-AP), Beta-Actin Antibody (Affinity, Cat.AF7018). Secondary antibody (HRP-conjugated Affinipure Goat Anti-Mouse IgG (H+L), Proteintech, Cat.SA00001-

1; HRP-conjugated Affinipure Goat Anti Rabbit IgG (H+L), Proteintech, Cat. SA00001-2)

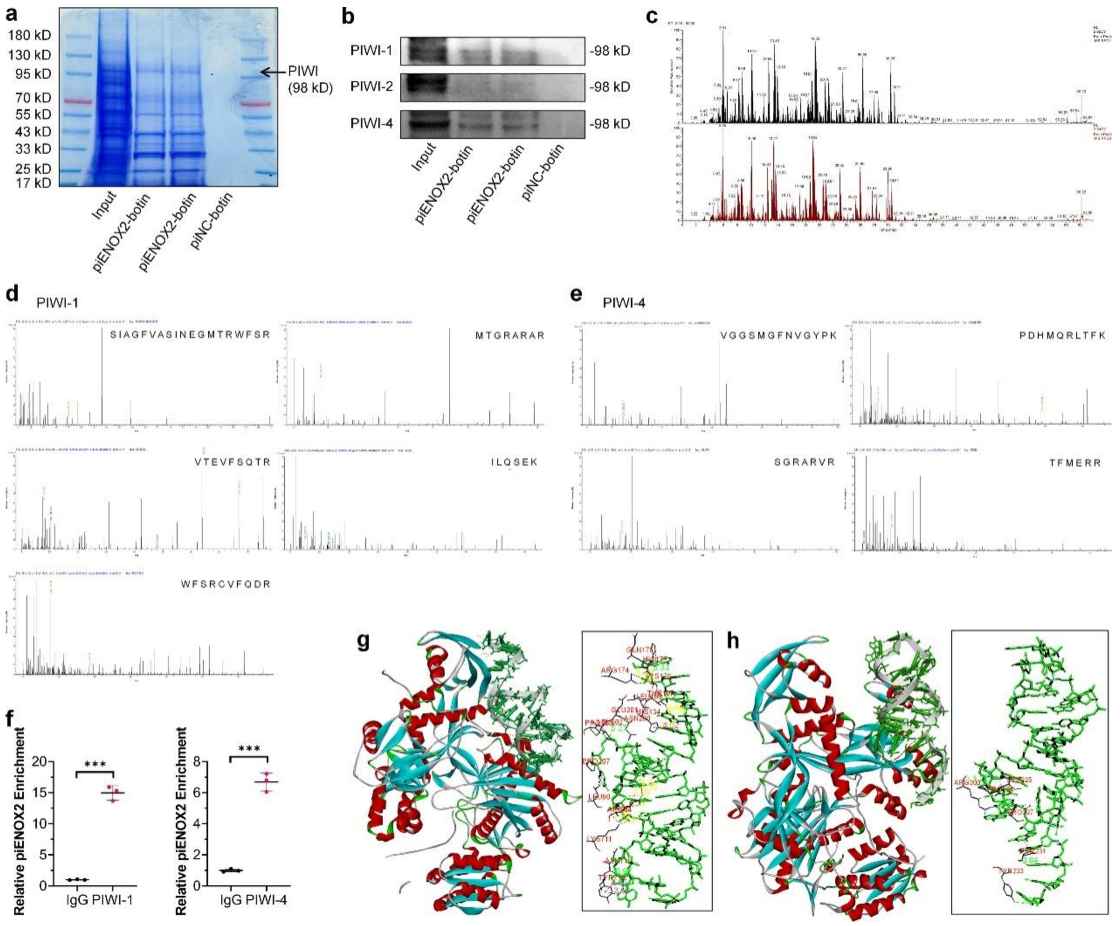

Supplementary Fig. 1 piENOX2 characterization. (a) Coomassie Brilliant Blue staining was used to observe the concentration and molecular weight of piENOX2-binding protein. (b) Western blot showing PIWI-1, PIWI-2, and PIWI-4 in piENOX2 binding proteins. (c) Protein spectrum analysis of piENOX2 binding proteins, (d) PIWI-1 and (e) PIWI-4 secondary mass spectrometry data of piENOX2 binding proteins. (f) RNA co-immunoprecipitation assessment of the expression of piENOX2 in RNA binding to PIWI-1 and PIWI-4. Molecular docking simulations of (g) PIWI-1 and (h) PIWI-4 binding sites with piENOX2. *** *P*<0.001.

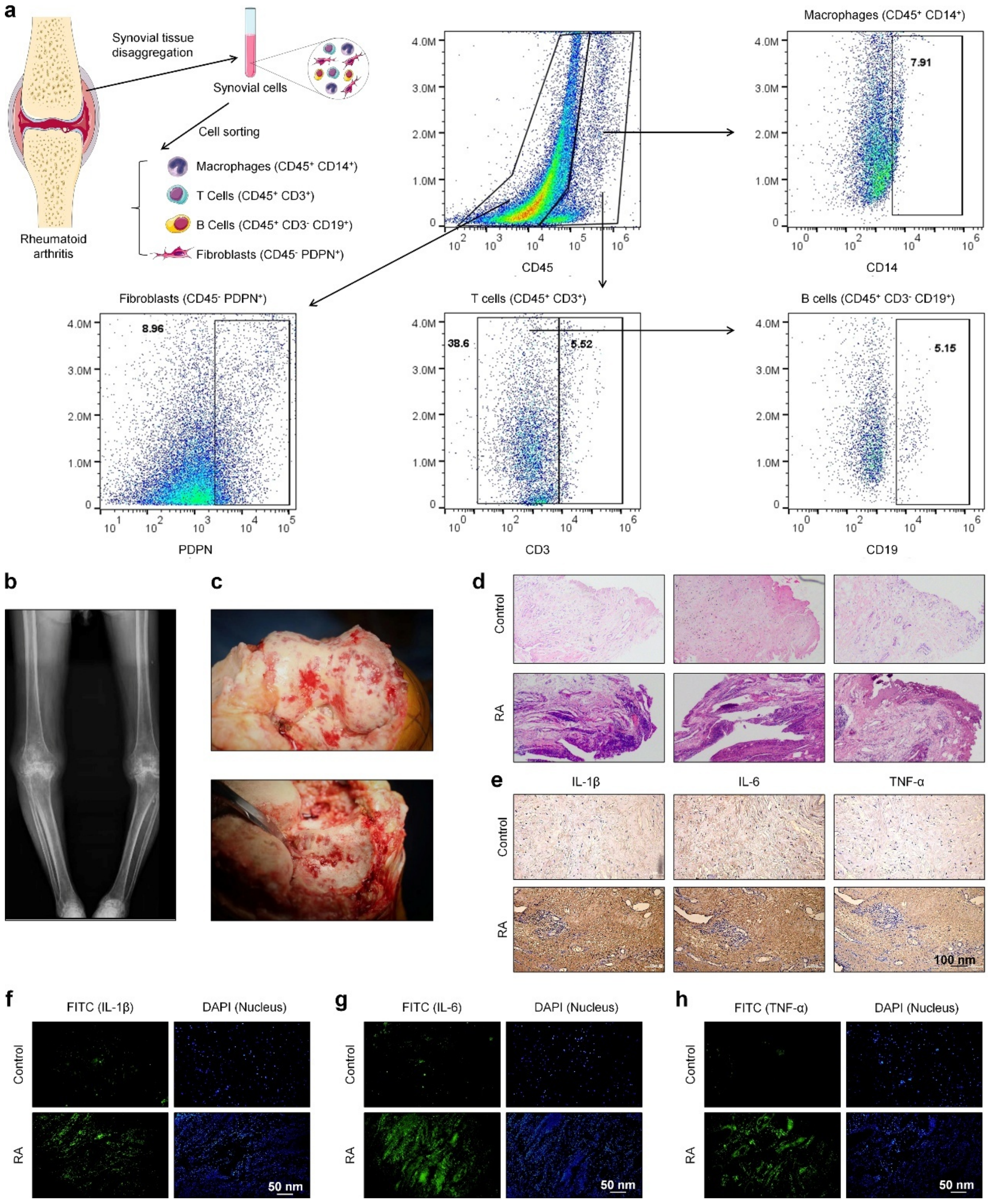

Supplementary Fig. 2 Expression and functional analysis of piENOX2. (a) Key cell populations in the synovial tissue, including synovial macrophages ($CD45^{+}CD14^{+}$), T cells ($CD45^{+}CD3^{+}$), B cells ($CD45^{+}CD3^{-}CD19^{+}$), and fibroblasts ($CD45^{-}PDPN^{+}$), were sorted and collected *via* flow cytometry. Synovial tissue samples from RA patients were collected. (b) X-ray image from an RA patient. (c) Intraoperative observation of synovial infiltration and cartilage damage in an RA patient. (d) HE staining of synovial

tissues of RA patients and controls. (e) Immunohistochemical detection of the expression and distribution of IL-1β, IL-6, and TNF-α in synovial tissue of RA patients and controls. (f-h) Immunofluorescence detection of IL-1β, IL-6, and TNF-α expression and distribution in synovial tissue of RA patients and controls.

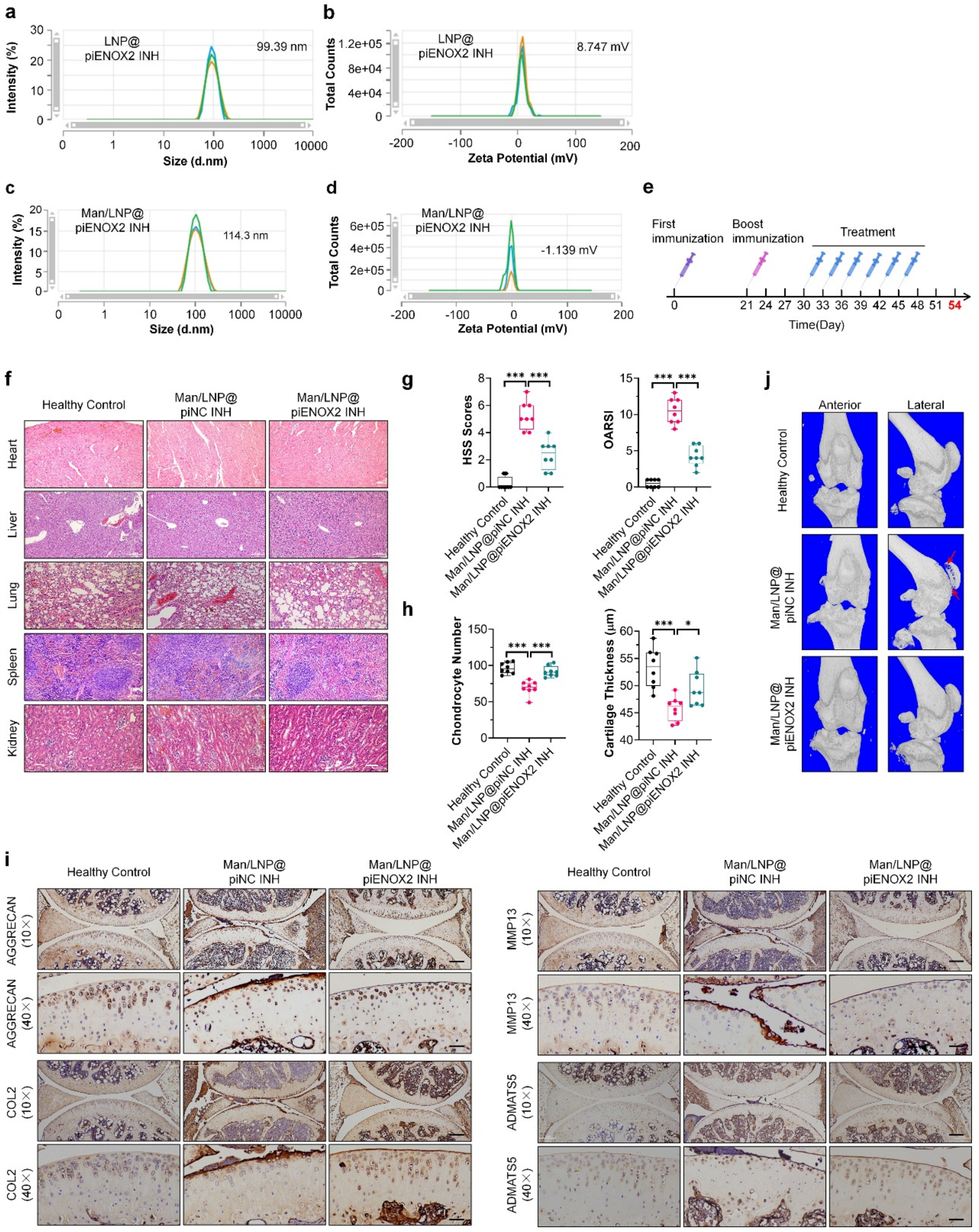

Supplementary Fig. 3 Man/LNP@piENOX2 INH effectively alleviates disease progression in CIA mice. (a) The particle size of the intermediate product LNP@piENOX2 INH measured by DLS. (b) Surface potential of the intermediate product LNP@piENOX2 INH. (c) Particle size of Man/LNP@piENOX2 INH measured by DLS. (d) Zeta potential of Man/LNP@piENOX2 INH. (e) Schematic

diagram of animal experiment schedule. (f) After treatment with different drugs, the heart, liver, lung, spleen and kidney tissues of mice in each group were collected at predetermined time points, and the histopathological conditions of each organ were observed following HE staining; scale bar = 100 μm. (g) The HE staining results of the affected knee joints of mice in each group were quantified through the HSS scoring system and the OARSI scoring system to evaluate synovial infiltration and cartilage damage. (h) Quantification of chondrocytes numbers and cartilage layer thickness of the affected knee joints of mice in each group using Safranin-O and Fast Green staining. (i) IHC showing the expression and distribution of markers related to cartilage synthesis and catabolism in the affected joint areas of each group of mice, with scale bars of 200 μm at 10× and 50 μm at 40× magnification. (j) Micro-CT images of the affected knee joint morphology and bone loss of mice in each group. $P^*<0.05$, $P^{**}<0.01$, $P^{***}<0.00$.

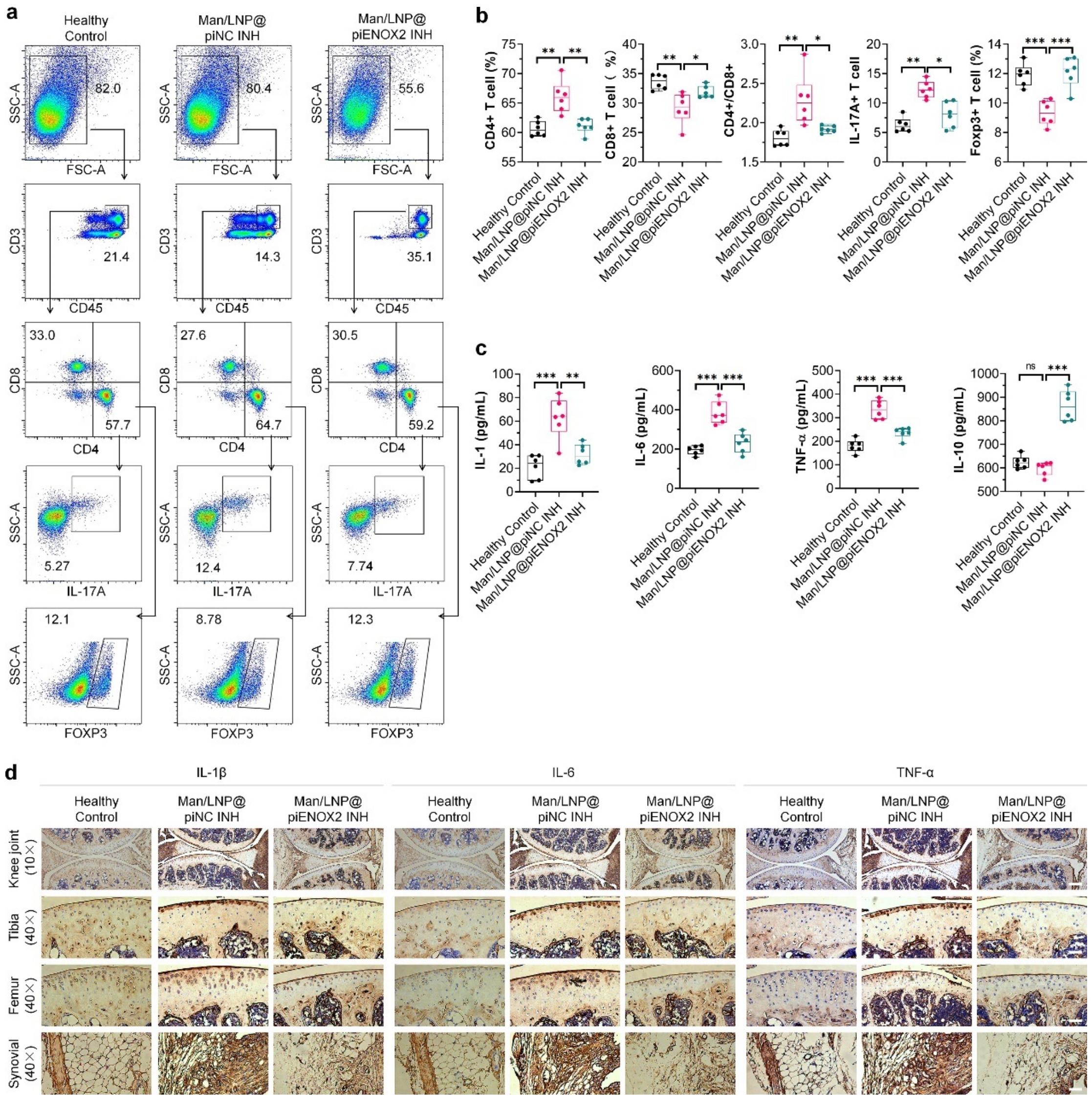

Supplementary Fig. 4 Man/LNP@piENOX2 INH maintains immune homeostasis and inhibits inflammation in CIA mice. (a-b) Flow cytometry was used to detect the proportion and ratio of $CD45^+CD3^+CD4^+$ T cells and $CD45^+CD3^+CD8^+$ T cells in each group, and the proportion of $CD45^+CD3^+CD4^+IL17A^+$ and $CD45^+CD3^+CD4^+FoxP3^+$ T cells ($N=6$). (c) ELISA measurements of the concentrations of pro-inflammatory cytokines IL-1β, IL6, TNF-α, and IL-10 in the serum of mice in each group ($N=4$). (d) IHC staining was used to observe the expression and distribution of pro-inflammatory cytokines IL-1β, IL-6, and TNF-α in the affected joint area. Scale bar in 10× = 200 μm and scale bar in 40× = 50 μm. $P^*<0.05$, $P^{**}<0.01$, $P^{***}<0.001$.

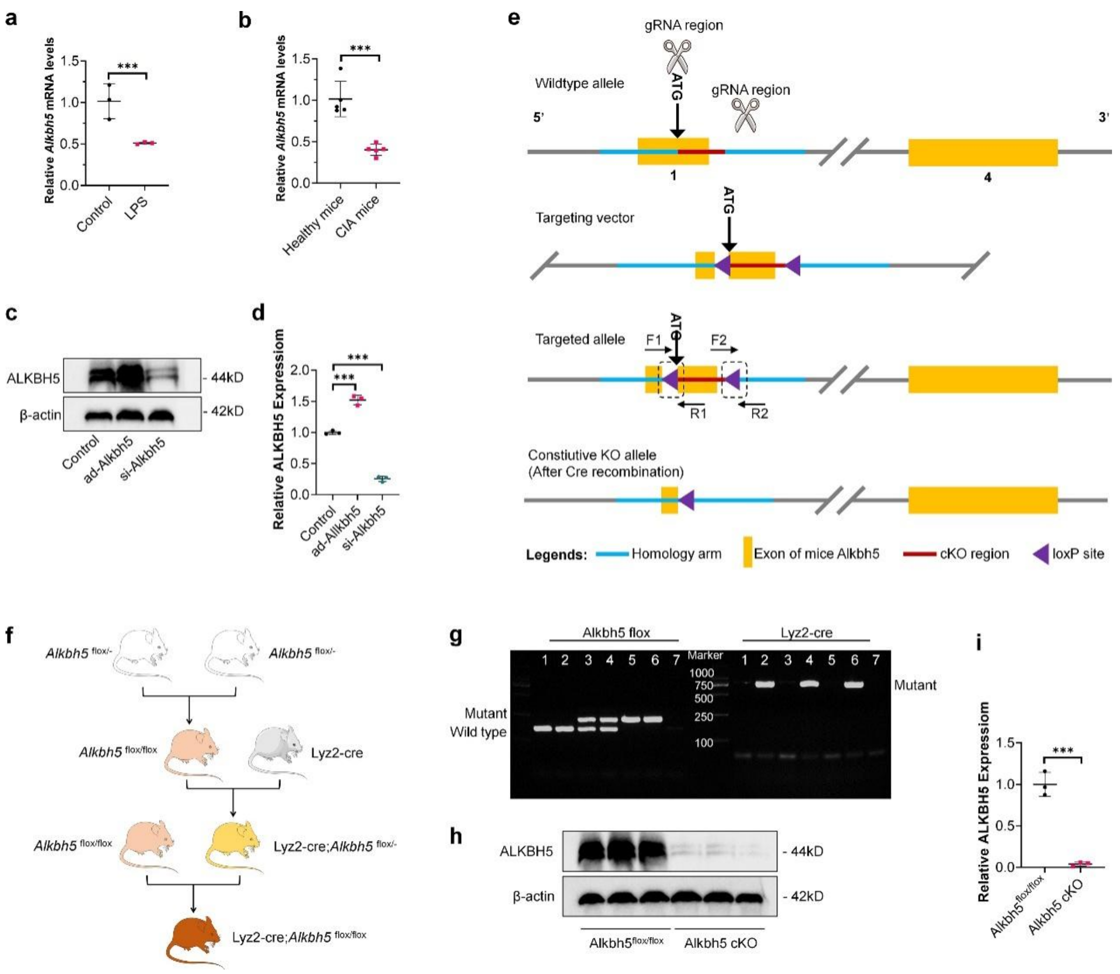

Supplementary Fig. 5 Verification of Alkbh5 adenovirus and siRNA efficiency and construction of Alkbh5-deficient mice. (a) RT-qPCR measurementof *Alkbh5* mRNA levels in an LPS-induced cell model (*N=3*). (b) RT-qPCR measurement of *Alkbh5* mRNA levels in CIA mice compared to control mice (*N=5*). (c-d) The overexpression efficiency of ad-Alkbh5 and the knockdown efficiency of si-Alkbh5 were verified in RAW 264.7 cells by western blottinh, with semi-quantification using ImageJ (*N=3*). (e) *Alkbh5* flox/- mice construction and (f) breeding scheme. (g) *Alkbh5* mouse identification results, 1, Wild type mice. 2, Lyz-cre+ mice. 3, *Alkbh5*$^{\text{flox/-}}$ mice. 4, *Alkbh5*$^{\text{flox/-}}$, Lyz-cre+ mice. 5, *Alkbh5*$^{\text{flox/flox}}$ mice. 6, *Alkbh5*$^{\text{flox/flox}}$, Lyz-cre+ mice. 7, Water. (h-i) The expression of ALKBH5 in primary BMDM cells of Alkbh5flox/flox

mice and Alkbh5 cKO mice was assessed by western blotting, with semi-quantification by ImageJ (*N=3*). *P****<0.001

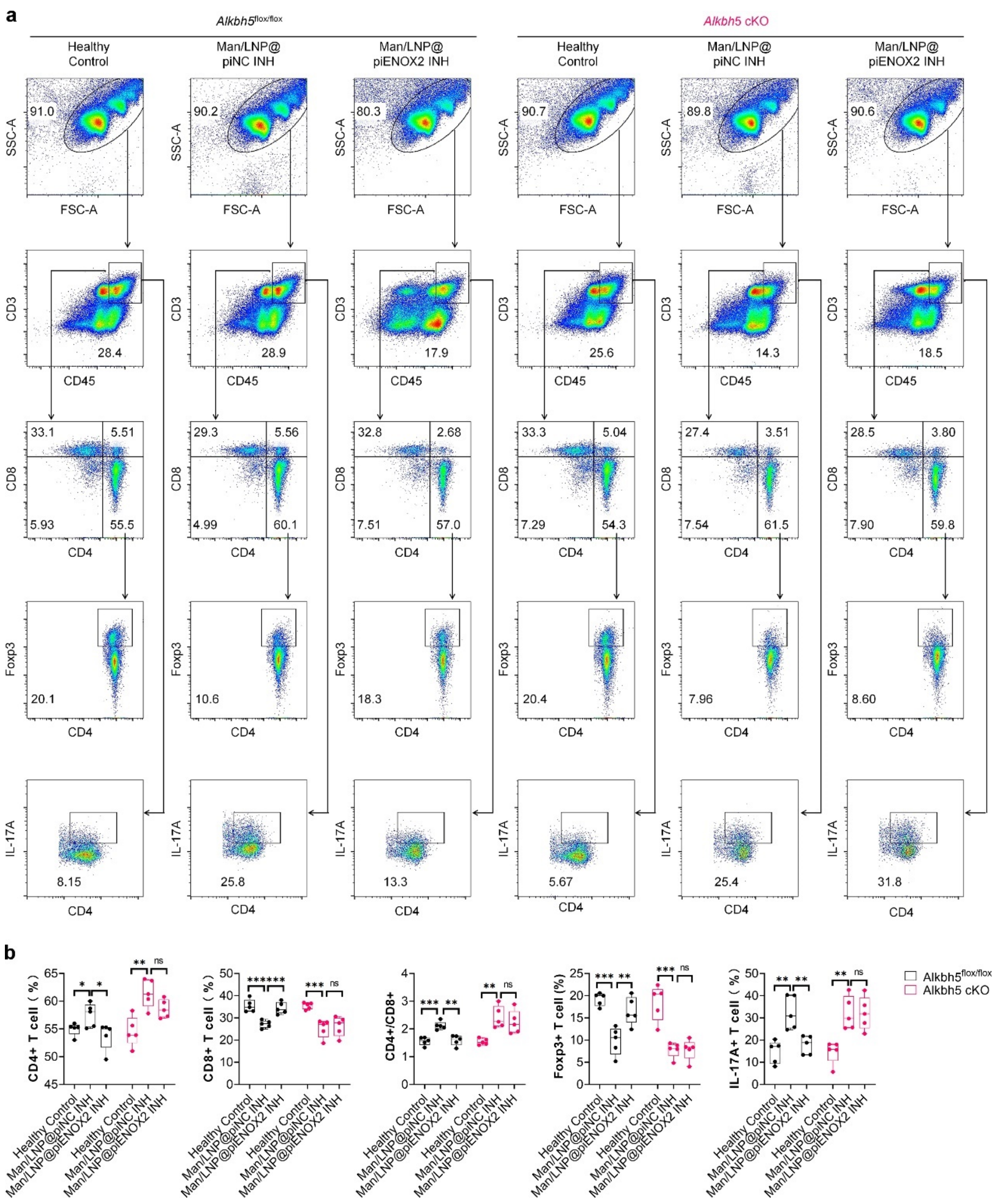

Supplementary Fig. 6 Knockout of *Alkbh5* blocks the effects of Man/LNP@piENOX2 INH in maintaining immune homeostasis and inhibiting inflammation in CIA model mice. (a-b) Flow cytometry was used to detect the proportion and ratio of $CD45^+CD3^+CD4^+$ T cells and $CD45^+CD3^+CD8^+$ T cells in each experimental mouse group, and the proportion of $CD45^+CD3^+CD4^+IL17A^+$ T and $CD45^+CD3^+CD4^+FoxP3^+$ T cells (N=5). ns, non-significant; P*<0.05, P**<0.01,

P***<0.001.

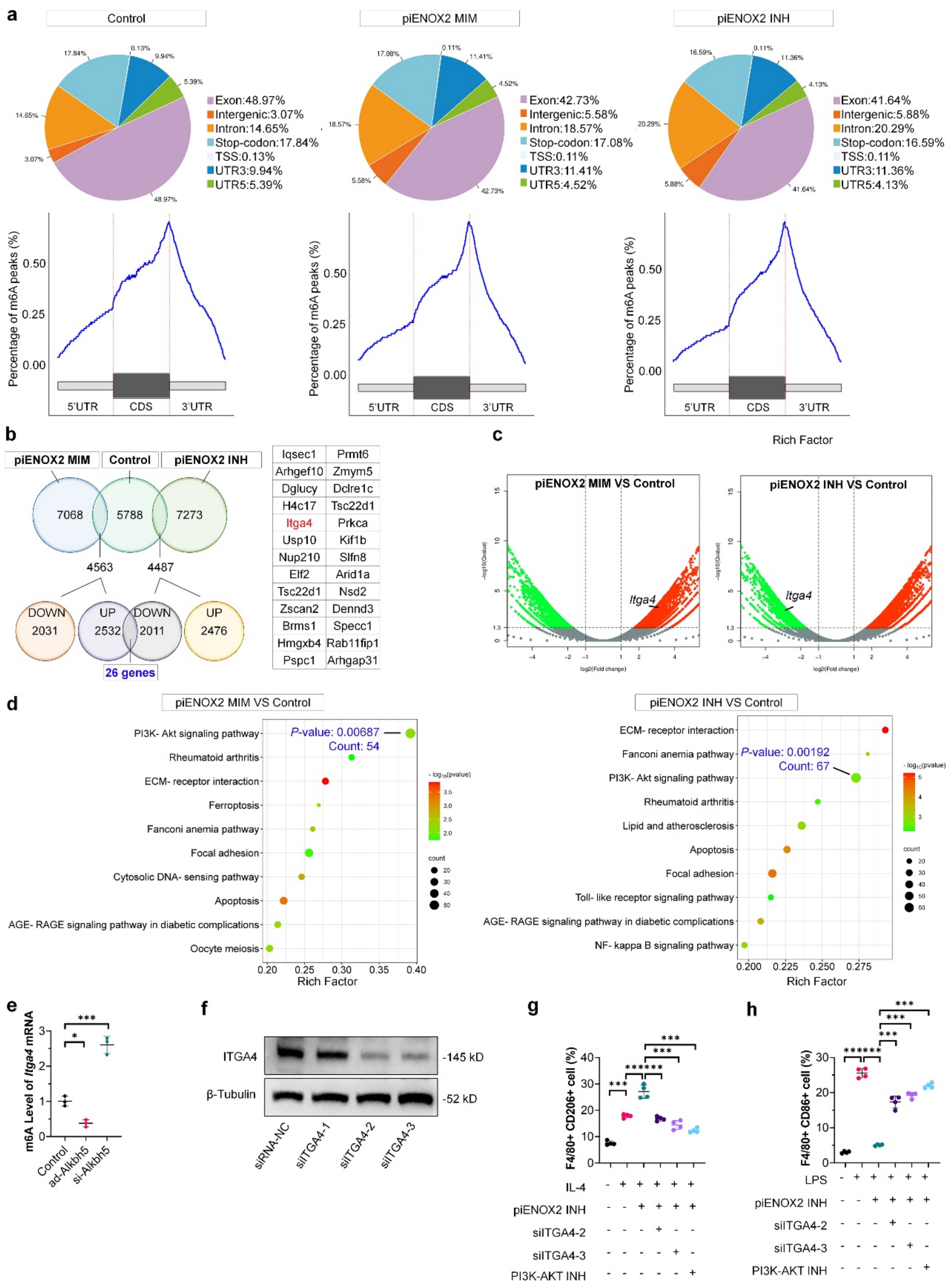

Supplementary Fig. 7 Supplementary data of meRIP-seq bioinformatics analysis and Itga4 siRNA screening. (a) In the LPS-induced inflammatory cell model, cells were treated with piENOX2 MIM and piENOX2 INH, and differentially methylated mRNA was detected and identified using meRIP-seq. (b) Based on these results,

differentially methylated mRNA genes were screened, identifying 26 genes that met the screening criteria. (c) piENOX2 mimics up-regulate *Itga4* m^6A levels, and piENOX2 inhibitor down-regulate *Itga4* m^6A levels. (d) KEGG results show that the PI3K/AKT signaling pathway is involved in piENOX2 regulating macrophage polarization. (e) *Itga4* siRNA validation, (f) semi-quantitative analysis was performed using ImageJ. (g) Flow cytometry analysis of the effects of *Itga4* knockdown or blocking the PI3K-AKT signaling pathway on piENOX2 INH-promoted M2 macrophage polarization (*N=4*). (h) Flow cytometry analysis of the effects of *Itga4* knockdown or blocking the PI3K-AKT signaling pathway on piENOX2 INH-inhibited M1 macrophage polarization (*N=4*). ***P <0.001.

Supplementary Table 1. Histological synovitis score (HSS) for assessing the morphological features of synovia.

Feature	Score
A. Hyperplasia or enlargement of synovial lining cell layer	
1. Absent	0
2. Slight enlargement (two to three cell layers). Giant cells are scarce	1
3. Moderate enlargement (four to five cell layers). Some giant cells or lymphocytes	2
4. Strong enlargement (more than six cell layers). Giant cells and lymphocytes are frequent	3
B. Inflammatory infiltration	
1. Absent	0
2. Slight inflammatory infiltration (diffusely located single cells and small perivascular aggregates of lymphocytes and/or plasma cells)	1
3. Moderate inflammatory infiltration (perivascular and/or superficial lymphatic aggregates and small–sized lymphatic follicles without germinal center may be observed)	2
4. Strong inflammatory infiltration (lymphatic follicles with germinal center and/or confluent subsynovial lymphatic infiltration)	3
C. Activation of synovial stroma/pannus formation	
1. Absent	0

2. Slight synovial stroma activation (low cellularity with slight edema, slight fibrosis with some fibroblast, no giant cells)	1
3. Moderate synovial stroma activation (moderate cellularity with a moderate density of fibroblasts, endothelial cells, and giant cells may be detected)	2
4. Strong synovial stroma activation (high cellularity with dense distribution of fibroblasts and endothelial cells, and giant cells are abundant)	3
Maximum possible score for grading	9

Supplementary Table 2. Modified Osteoarthritis Research Society International (OARSI) scores for microscopic evaluation of cartilage status.

Feature	Score
A. Structure	
0. Normal	0
1. Slight surface irregularities	1
2. Moderate surface irregularities	2
3. Severe surface irregularities	3
4. Clefts/fissures into transitional zone (one−third depth)	4
5. Clefts/fissures into radial zone (two−thirds depth)	5
6. Clefts/fissures into calcified zone (full depth)	6
7. Fibrillation and/or erosion to transitional zone (one−third depth)	7
8. Fibrillation and/or erosion to radial zone (two−thirds depth)	8
9. Fibrillation and/or erosion to calcified zone (full depth)	9
10. Fibrillation and/or erosion to subchondral bone	10
B. Cellularity	
0. Normal	0
1. Increase or slight decrease	1
2. Moderate decrease	2
2. Moderate decrease	3
4. No cells present	4

C. Chondrocyte cloning	
0. Normal	0
1. Several doublets	1
2. Many doublets	2
3. Doublets and triplets	3
4. Multiple cell nests	4

Supplementary Table 3. siRNA sequences.

Name	Sequence
Alkbh5 siRNA target sequence	GCATACGGCCTCAGGACAT
Itga4 siRNA-2 sense	GCAUCAUGUCAAGCUGGAA
Itga4 siRNA-2 antisense	UUCCAGCUUGACAUGAUGC
Itga4 siRNA-3 sense	GAGUAUUCGUGUACAUCAA
Itga4 siRNA-3 antisense	UUGAUGUACACGAAUACUC

Supplementary Table 4. RT-qPCR primer sequences.

Name	Sequence
M-*Gapdh*-F	AGGTCGGTGTGAACGGATTTG
M-*Gapdh*-F	TGTAGACCATGTAGTTGAGGTCA
H-*Gapdh*-F	TGTGTCCGTCGTGGATCTGA
H-*Gapdh*-F	TTGCTGTTGAAGTCGCAGGAG
M-*Alkbh5*-F	CGCGGTCATCAACGACTACC
M-*Alkbh5*-R	ATGGGCTTGAACTGGAACTTG
H-*Alkbh5*-F	CGGCGAAGGCTACACTTACG
H-*Alkbh5*-R	CCACCAGCTTTTGGATCACCA
H-*Il-1β*-F	AGCTACGAATCTCCGACCAC
H-*Il-1β*-R	CGTTATCCCATGTGTCGAAGAA
M-*Il-1β*-F	GCAACTGTTCCTGAACTCAACT
M-*Il-1β*-R	ATCTTTTGGGGTCCGTCAACT
H-*Il-6*-F	ACTCACCTCTTCAGAACGAATTG
H-*Il-6*-R	CCATCTTTGGAAGGTTCAGGTTG
M-*Il-6*-F	TAGTCCTTCCTACCCCAATTTCC
M-*Il-6*-R	TTGGTCCTTAGCCACTCCTTC
H-*Tnf-α*-F	CCTCTCTCTAATCAGCCCTCTG
H-*Tnf-α*-R	GAGGACCTGGGAGTAGATGAG
M-*Tnf-α*-F	CCCTCACACTCAGATCATCTTCT

M-*Tnf-α*-R	GCTACGACGTGGGCTACAG
M-*Cd206*-F	CTCTGTTCAGCTATTGGACGC
M-*Cd206*-R	CGGAATTTCTGGGATTCAGCTTC
M-*Arg1*-F	CTCCAAGCCAAAGTCCTTAGAG
M-*Arg1*-R	AGGAGCTGTCATTAGGGACATC
M-*iNos*-F	GTTCTCAGCCCAACAATACAAGA
M-*iNos*-R	GTGGACGGGTCGATGTCAC
M-*Cd86*-F	TGTTTCCGTGGAGACGCAAG
M-*Cd86*-R	TTGAGCCTTTGTAAATGGGCA
H-*Itga4*-F	TACAGATGCAGGATCGGAAAGA
H-*Itga4*-R	AGGTTCTCCATTAGGGCTACC
M-*Itga4*-F	GATGCTGTTGTTGTACTTCGGG
M-*Itga4*-R	ACCACTGAGGCATTAGAGAGC

Abbreviations: H, human; M, mouse; F, forward primer; R, reverse primer.

REFERENCES

1. Inglis, J.J., Simelyte, E., McCann, F.E., Criado, G. & Williams, R.O. Protocol for the induction of arthritis in C57BL/6 mice. *Nat. Protoc.* **3**, 612-618 (2008).
